# A dual-signals peptide-based probe for fluorometric and colorimetric detection of copper(II) ions and glyphosate in multiple food samples and biological system

**DOI:** 10.1016/j.fochx.2026.103776

**Published:** 2026-03-21

**Authors:** Yi Ren, Mengying Jia, Shiyi Xiong, Yong An, Xiupei Yang, Peng Wang

**Affiliations:** a Precise Synthesis and Function Development Key Laboratory of Sichuan Province, College of Chemistry and Chemical Engineering, China West Normal University, Shida Road 1#, Nanchong 637009, PR China; b The First School of Clinical Medicine, Gansu University of Chinese Medicine, Lanzhou, Gansu 730030, PR China

**Keywords:** Dual-signals peptide-based probe, Colorimetric and fluorometric sensing, Copper(II) ions, Glyphosate, Multifunctional application

## Abstract

A new dual-signals peptide-based probe **FAHK** was rationally designed and successfully synthesized for sequential detection of Cu^2+^ and glyphosate through fluorescence and colorimetric sensing method. The limit of detections (LODs) for Cu^2+^ and glyphosate were calculated to be 49.7 nM and 66.3 nM, respectively. Additionally, **FAHK** not only demonstrated excellent accuracy in the determination of real food, water and vegetables samples, but also was applied for fluorescence imaging Cu^2+^ and glyphosate in two biological systems. Besides, test strips experiments showed that **FAHK** has extraordinary potential in environmental monitoring and food analysis applications. Meanwhile, we explored the application of **FAHK** in molecular logic gates, and a visual portable sensing platform was established *via* the smartphone color picker *App* system, and successfully achieving semi-quantitative analysis of Cu^2+^ and glyphosate. More importantly, **FAHK** has successfully achieved the detection of glyphosate residues on six food surfaces and soil sample with satisfactory results.

## Highlights

• Dual-signals peptide-based probe **FAHK** was fabricated for continuous detection of Cu^2+^ and glyphosate.
• The detection limit of 49.7 nM for Cu^2+^ and 66.3 nM for glyphosate.
• **FAHK** was applied for determination of Cu^2+^ and glyphosate in biological system and food samples.
• The practical utility **FAHK** was studied using test strips, logic gate and smartphone *App*.
• **FAHK** achieved naked-eye detection of Cu^2+^ and glyphosate residues on surfaces of food and soil.

## Graphical abstract

**Figure:**
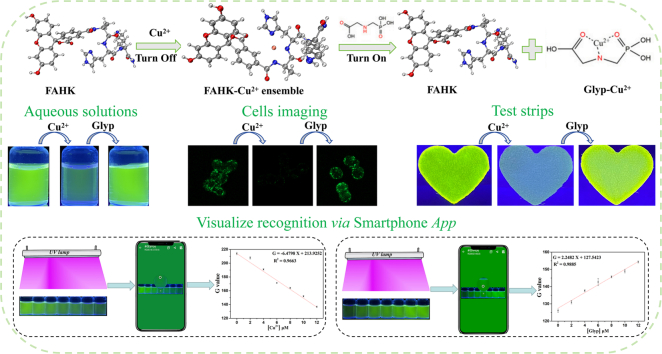

## 1 Introduction

Food safety has received extensive attention due to its significant impact on human health (Hordyjewska et al., 2014; Li et al., 2022). Copper ions (Cu^2+^) is a necessary trace element that plays multiple important physiological roles in the human body. Cu^2+^ not only constitute a part of many enzymes, but are also crucial for the development and function of nerve cells (Che et al., 2024; Hao et al., 2024; Li et al., 2022; Wang, Wang, et al., 2025). In addition, Cu^2+^ also have the functions of catalyzing the synthesis of hemoglobin, preventing cardiovascular diseases, promoting bone development, reducing inflammation and rheumatism, et al. (Bisaglia & Bubacco, 2020; Che et al., 2024; Karakiliç et al., 2025; Zhao et al., 2025). However, excessive Cu^2+^ has adverse effects on the nervous system, liver and blood system, etc. (Bisaglia & Bubacco, 2020; Chen et al., 2025; Hordyjewska et al., 2014; Liu et al., 2025; Zhao et al., 2025). Glyphosate (Glyp), as a non-selective and residue-free systemic herbicide, has currently the most widely used and largest-yielding agricultural chemical in the world due to its advantages such as high efficiency, low toxicity and broad-spectrum coverage (Conrad et al., 2017; Guan et al., 2023; Li et al., 2022). However, the excessive residues of glyphosate can pose a threat to human health, causing damage to organs such as the respiratory tract, digestive tract, and skin mucous membranes (Li, Jiang, et al., 2025; Liang et al., 2024; Peng et al., 2025; Rong et al., 2025; Yang et al., 2024). In severe cases, glyphosate can also harm the respiratory and cardiovascular systems, and even endanger life (Li, Jiang, et al., 2025; Liang et al., 2024; Peng et al., 2025; Rong et al., 2025; Zhan et al., 2025). Based on this, glyphosate has classified as a 2 A class carcinogen by the World Health Organization (WHO) (Qin et al., 2025; Zhan et al., 2025; Zhang et al., 2024). In over 30 countries and regions around the world, the use of glyphosate has been banned or restricted, and monitoring of glyphosate residues has been strengthened (He et al., 2024; Zhan et al., 2025). Therefore, it has significant value to develop a detection method with excellent characteristics such as easy operation, rapid response, high sensitivity, and wide applicability for Cu^2+^ and glyphosate detection in real samples and ecosystems.

Compared with other detection methods, fluorescence sensing has been widely applied in the highly selective detection of Cu^2+^ and glyphosate due to its advantages of simple operation, strong specificity, fast response and low cost (AbhijnaKrishna & Velmathi, 2022; Fu et al., 2025; Li et al., 2022; Liu et al., 2020; Tian et al., 2025; Zhai et al., 2025). Furthermore, fluorescent probes are particularly suitable for conducting fluorescence imaging studies in biological systems (AbhijnaKrishna & Velmathi, 2022; Li et al., 2022). The colorimetric detection method is a simple and efficient analytical approach established based on the naked-eye observation of fluorescence brightness and color, which has the unique advantages of being simple and easy to operate, high sensitivity, good selectivity, and wide applicability (AbhijnaKrishna & Velmathi, 2022; Alanazi et al., 2025; Wang et al., 2020; Wang, Li, et al., 2025; Wang, Zheng, et al., 2025; Zheng et al., 2024), so it is widely used in various fields for the visual determination of trace components. To date, many fluorescent probes have been reported for the detection of Cu^2+^ and glyphosate (AbhijnaKrishna & Velmathi, 2022; Che et al., 2024; Chen et al., 2025; Hao et al., 2024; Li et al., 2022; Zhan et al., 2025; Zhao et al., 2025; Zheng et al., 2024). However, many organic small molecule fluorescent probes have disadvantages such as poor water solubility, low sensitivity, and relatively low binding affinity. Some nano-probes have complex and risky synthesis processes and due to high toxicity, there is a risk of secondary pollution in the detection process. These obvious deficiencies severely restrict their efficient application in food samples and biological systems (Table S7 and Table S8). Therefore, developing fluorescent and colorimetric dual-signals probe with excellent water solubility and low toxicity for of Cu^2+^ and glyphosate detection is of great significance.

In recent years, many research teams have successively reported many fluorescent probes based on peptide receptors due to many unique advantages, such as (1) peptide-based probes can be efficiently synthesized through peptide solid-phase synthesis (SPPS) technology and fluorescence modification technology (An et al., 2024; Jia et al., 2026; Subedi et al., 2025; Wang et al., 2021). (2) peptide molecule has excellent water solubility, cell permeability and low toxicity (Jung & Lee, 2015; Li et al., 2026; Wang, Li, et al., 2025; Zheng et al., 2024). (3) The amino acid residues of specific peptides can bind to heavy metal ions with strong affinity (Li et al., 2024; Pu et al., 2024; Wang et al., 2020; Wang, Zheng, et al., 2025; Xu et al., 2019). Considering the binding mode of the reported probes based on peptides, we developed a new dual-signals peptidyl probe **FAHK** with fluorescent and colorimetric by coupling 5-carboxy fluorescein (5-FAM) labeled tripeptide (Ala-His-Lys-NH_2_). **FAHK** featured high selectivity (only Cu^2+^), fast response (within 25 s), low detection limit (49.7) and wide pH application range (2−12) for Cu^2+^ detection. In addition, the non-fluorescence **FAHK**-Cu^2+^ ensemble has many advantages for detection of glyphosate as a new promising cascade probe. Notably, **FAHK** achieved many application in biological systems, real food and water samples, test strips, and NHIBIT molecular logic gate, showing promising practical prospects. In addition, smartphone-assisted **FAHK** visual portable sensing platform facilitated visual analysis of Cu^2+^ and glyphosate with satisfactory results. More importantly, **FAHK** was further successfully utilized to image the residues of glyphosate in six food samples, as well as applied for monitoring glyphosate degradation in soil sample.

## 2 Experimental section

### 2.1 Materials and apparatus

The main materials for peptide synthesis were purchased from Top-peptide Co., Ltd. (Shanghai, China). All raw materials and reagents used in the synthesis and testing were purchased from commercial suppliers from Heowns Biochem Technologies, LLC. (Tianjin, China), and no further purification of the chemicals was performed. All metal ions, anions and pesticides were prepared distilled water. All testing experiments were conducted using 100% aqueous solution containing 10 mM HEPES buffer at pH 7.4.

The mass spectrum was obtained using Bruker Daltonics Esquire 6000 spectrometer (Bruker Daltonics, Germany). High resolution mass (HRMS) spectra was employed on Thermo Scientific Q Exactive (Thermo Fisher Scientific, United States). ^1^H HRMS was performed with a Bruker 400 MHz instruments in DMSO‑*d*_6_ (Bruker Daltonics, Germany). The fluorescence emission spectrum was recorded with Gangdong F-380 fluorescence spectrophotometer (Gangdong, China). UV–Vis absorption spectra were recorded on a Varian UV-Cary100 spectrophotometer in the range of 200–800 nm (Varian, United States).

### 2.2 Synthesis and characterization

**Scheme 1:**
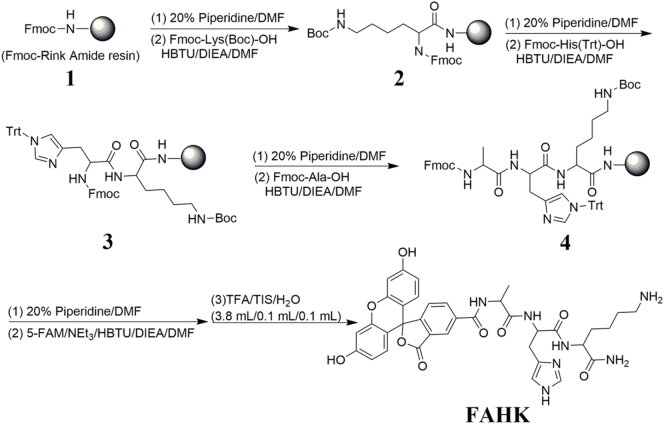
Schematic illustration of the synthesis of probe **FAHK**.

The synthetic route used for probe **FAHK** according to the previous reports (Scheme 1). Firstly, the tripeptide (Ala-His-Lys-NH_2_) was successfully synthesized using solid-phase peptide synthesis (SPPS) method. Then, 5-carboxy fluorescein (5-FAM) was coupled with the tripeptide in DMF solution at room temperature for 4 h. After cleavage from the resin by treatment with a mixture of 4 mL TFA: TIS: H_2_O (95:2.5:2.5, *v*/v/v) for 3 h. The high purity (95.43%) of **FAHK** was analyzed using an analytical HPLC on a C18 analytical column (Fig. S1 and Table S1). ESI-MS (*m*/*z*): [M + H^+^]^+^ calculated for C_36_H_37_N_7_O_9_: 711.70, observed: 712.61 (Fig. S2). ^1^H NMR (400 MHz, DMSO‑*d*_6_) *δ* 14.32 (d, *J* = 30.8 Hz, 1H), 10.24 (s, 1H), 9.01 (t, *J* = 3.4 Hz, 1H), 8.55 (d, *J* = 1.6 Hz, 1H), 8.41 (d, *J* = 8.0 Hz, 1H), 8.29 (dd, *J* = 8.0, 1.6 Hz, 1H), 8.04 (d, *J* = 7.6 Hz, 1H), 7.90 (s, 2H), 7.54 (s, 1H), 7.49–7.28 (m, 2H), 7.26–7.02 (m, 1H), 6.72 (d, *J* = 1.8 Hz, 1H), 6.57 (d, *J* = 1.9 Hz, 3H), 4.70–4.56 (m, 1H), 4.48 (t, *J* = 7.0 Hz, 1H), 4.22–4.11 (m, 1H), 3.31–2.91 (m, 4H), 2.75 (s, 2H), 2.33 (s, 1H), 2.05–1.91 (m, 1H), 1.81–1.45 (m, 3H), 1.45–1.13 (m, 5H), 0.84 (d, *J* = 7.1 Hz, 1H) (Fig. S3).

### 2.3 Ethical statement, cell and zebrafish larvae cultivation

Mouse prostate cancer cells (RM1 cells) were purchased from Promesa Biotechnology company (Wuhan, China), and the zebrafish larvae were purchased from Ezerinka company (Nanjing, China). All procedures stated in this work comply with the relevant laws and institutional guidelines. Animal procedures were executed following Guidelines for Care and Use of Laboratory Animals of Gansu University of Chinese Medicine and were approved by Animal Ethics Committee of Gansu University of Chinese Medicine, China. All the experiments were performed and approved under the Guidelines for the Care and Use of Laboratory Animals: Eighth Edition (ISBN10:0–309–15,396–4). RM1 cells were cultured in DMEM supplemented with 10% fetal bovine serum (FBS) and 1% antibiotic-antimycotic solution. Zebrafish maintenance and embryo collection were performed according to standard operating procedures as described elsewhere. Cells were maintained at 37 °C in a humidified atmosphere containing 5% CO_2_. The collected embryos were washed using standard zebrafish E3 culture medium (5 mmol L^−1^ NaCl, 0.33 mmol L^−1^ CaCl_2_, 0.33 mmol L^−1^ MgSO_4_·7H_2_O, 0.17 mmol L^−1^ KCl) at the one-cell to two-cell stage, and then incubated at 28.5 °C in E3 culture medium.

### 2.4 Measurement in real samples

Watermelon juice, apple juice, and hami melon juice were selected in order to determine Cu^2+^ and glyphosate content of food samples. The three samples were weighed and subjected to ultrasonic treatment. Then, the mixture was centrifuged and the supernatant was extracted. The same method was used to prepare the test solution for soil samples (Li, Meng, et al., 2025; Zhou et al., 2025).

## 3 Results and discussion

### 3.1 Spectral response of **FAHK** towards Cu^2+^

**Fig. 1:**
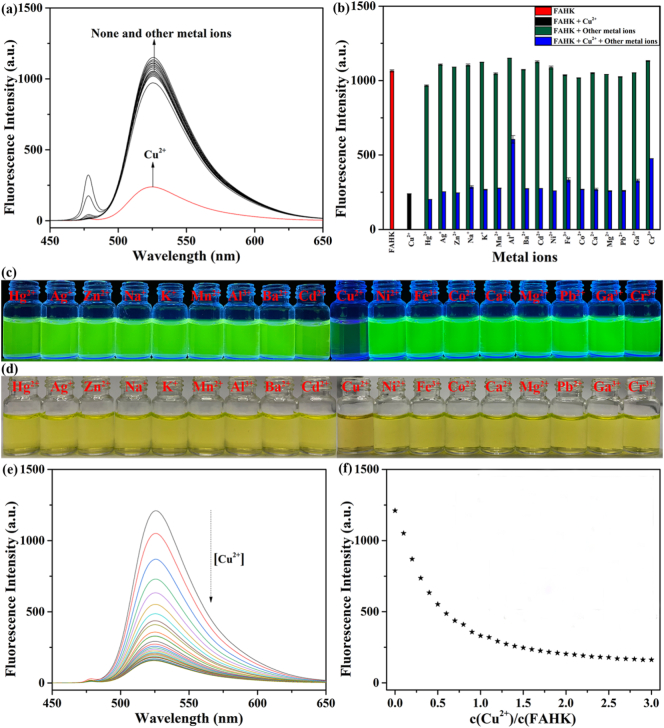
(a) Fluorescence selectivity experiment of **FAHK** (10 μM) in the presence of various metal ions (10 μM). (b) Fluorescence anti-interference experiment of **FAHK** (10 μM) with Cu^2+^ (10 μM) in the presence of competitive metal ions (50 μM). Visible color change of **FAHK** towards metal ions under 365 nm UV light (c) and natural light (d). (e) Fluorescence spectra of **FAHK** (10 μM) in the presence of different Cu^2+^ concentrations (0–30 μM). (f) Linear relationship between the fluorescence intensity and Cu^2+^ concentration.

To better verify the probe's selectivity, the fluorescence selective experiment of **FAHK** were carried out in the presence of various metal ions. Only the addition of Cu^2+^ caused a significant quenching change in the fluorescence spectrum of **FAHK**. It was noteworthy that **FAHK** has only weak fluorescence signals in the presence of other metal ions (Fig. 1a). In addition, the UV–Vis spectra of **FAHK** in the presence of different metal ions were carried out. After adding Cu^2+^, the absorption peak of **FAHK** were obviously weakened at 495 nm, and the absorption peak at 225 nm significantly enhanced. Fortunately, no significant UV–vis absorption signal changes observed from the other metal ions under the same spectral conditions (Fig. S4). These experimental results fully demonstrated that **FAHK** exhibited excellent selectivity for Cu^2+^ and enables specific detection of Cu^2+^. Competitive experiment result demonstrated that the presence of excessive other metal ions showed no drastic interference effect during the process of detecting Cu^2+^ (Fig. 1b). Additionally, after adding Cu^2+^ to **FAHK** solution, the bright green fluorescence basically disappeared completely under UV light at 365 nm (Fig. 1c), and the selective colorimetric response from greenish-yellow to dark yellow towards Cu^2+^ also observed using the naked eye under natural light (Fig. 1d).

The sensitivity of **FAHK** towards Cu^2+^ was further performed through fluorescence titration experiment. Increasing Cu^2+^ concentrations (0–30 μM) were gradually added to **FAHK** solution (10 μM), the fluorescence peak of **FAHK** at 525 nm was gradually decreased (Fig. 1e), and the fluorescence intensity reached a plateau when Cu^2+^ concentration approached 20 μM (Fig. 1f). In addition, the corresponding CIE chromaticity coordinated precisely corresponded the fluorescence titration data (Fig. S5). Moreover, we inspected the changes in UV–vis absorption spectra of **FAHK** after the introduction of varying concentrations of Cu^2+^, the absorption peak of **FAHK** gradually weakened at 495 nm, while gradually strengthened at 218 and 240 nm. Moreover, an isosbestic point appeared at 400 nm, which demonstrated that Cu^2+^ have formed stable complexes with **FAHK** (Fig. S6). Job's plot analysis indicated that 1:1 complexation stoichiometry between **FAHK** and Cu^2+^ based on a distinct maximum at a mole fraction of 0.5 (Fig. S7). Furthermore, the ESI high-resolution mass spectrometry (ESI-HRMS) of the **FAHK**-Cu^2+^ ensemble showed an *m*/*z* peak at 773.21848, corresponding to the involvement of [**FAHK** + Cu^2+^ − 2H^+^], which revealed that a 1:1 ligand-to-metal binding ratio for the **FAHK**-Cu^2+^ ensemble (Fig. S8). Subsequently, ^1^H NMR titrations of **FAHK** with Cu^2+^ was performed in DMSO‑*d*_6_ (Fig. S9). With the addition of Cu^2+^, many proton peaks in 8.50⁓7.50 ppm range also gradually weakened, indicating that the N atom of the imino group from tripeptide (Ala-His-Lys-NH_2_) was involved in the coordination with Cu^2+^. In addition, the proton of other functional groups have little change. Furthermore, the FTIR spectroscopy of **FAHK** with Cu^2+^ was measured (Fig. S10). By comparison, one peak at 3408 disappeared in the FTIR spectroscopy when the Cu^2+^ and **FAHK** formed the **FAHK**-Cu^2+^ ensemble, and we speculated that they correspond to the out-of-plane bending N—H peak on imine group. The peak at 1685 moved to about 1350, which may be caused by the coordination between N on imidazole from histidine and Cu^2+^, resulting in the movement of the C—H extended peak. In addition, the disappearance of other FTIR spectral peaks can be attributed to the paramagnetic quenching effect of Cu^2+^. The results indicated that **FAHK** forms a stable complex with Cu^2+^ *via* one N atoms on the imidazole group from histidine and and two N atoms on imine groups from alanine. Moreover, the binding affinity constant of the **FAHK**-Cu^2+^ ensemble was calculated to 1.9 × 10^5^ M^−1^ from the Benesi-Hilderbrand Plot (R^2^ = 0.9890), indicating that a stronger binding between **FAHK** and Cu^2+^ (Fig. S11). The detection limit (LOD) of **FAHK** for Cu^2+^ calculated as 49.7 nM according to formula LOD = 3σ/*k* (Fig. S12).

**Fig. 2:**
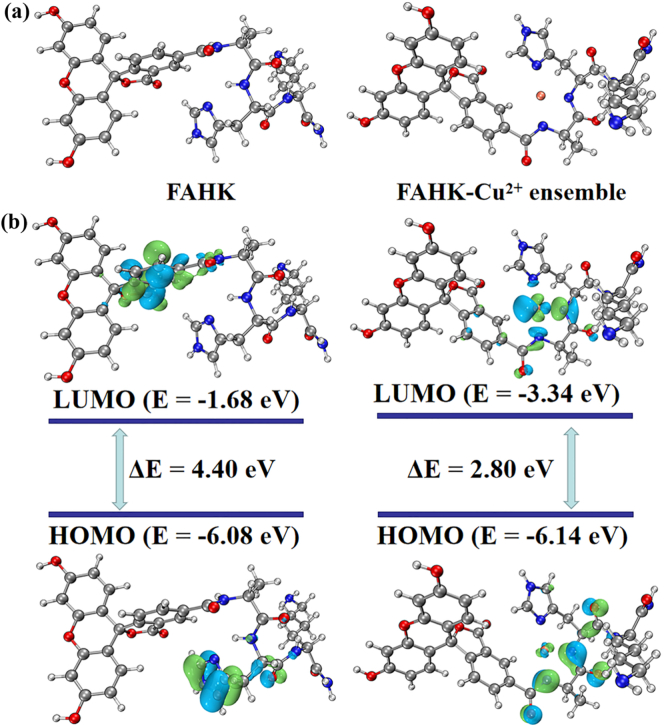
(a) Optimized geometric structures of **FAHK** and the **FAHK**-Cu^2+^ ensemble. (b) Frontier molecular orbital distributions of **FAHK** and the **FAHK**-Cu^2+^ ensemble obtained from DFT calculations.

To further confirm the specific recognition mechanism of **FAHK** for Cu^2+^, the density functional theory were studied by using Gaussian16, A03 software package (Frisch et al., 2009). The optimization binding modes and the computed molecular structure of **FAHK** and **FAHK**-Cu^2+^ was depicted in Fig. 2a. Subsequently, the band gap energy of **FAHK** and **FAHK**-Cu^2+^ ensemble were also calculated from the HOMO and LUMO energy levels (Lu & Chen, 2012). The HOMO of **FAHK** was mainly located in the histidine part, while the LUMO was positioned on the 5-carboxyl fluorescein. However, when **FAHK** forms a coordination with Cu^2+^, the distribution of the electron cloud has significantly shifted, and the orbital overlap between HOMO and LUMO also significantly increased (Fig. 2b). Moreover, the HOMO-LUMO gap value was calculated as 4.40 eV. Upon coordination with Cu^2+^, the HOMO-LUMO gap markedly decreased to 2.80 eV, indicating that the reduction of the HOMO-LUMO gap promoted the increase of charge transfer between **FAHK** and Cu^2+^, thereby forming a stable complex.

### 3.2 Spectral response of **FAHK**-Cu^2+^ ensemble towards glyphosate

**Fig. 3:**
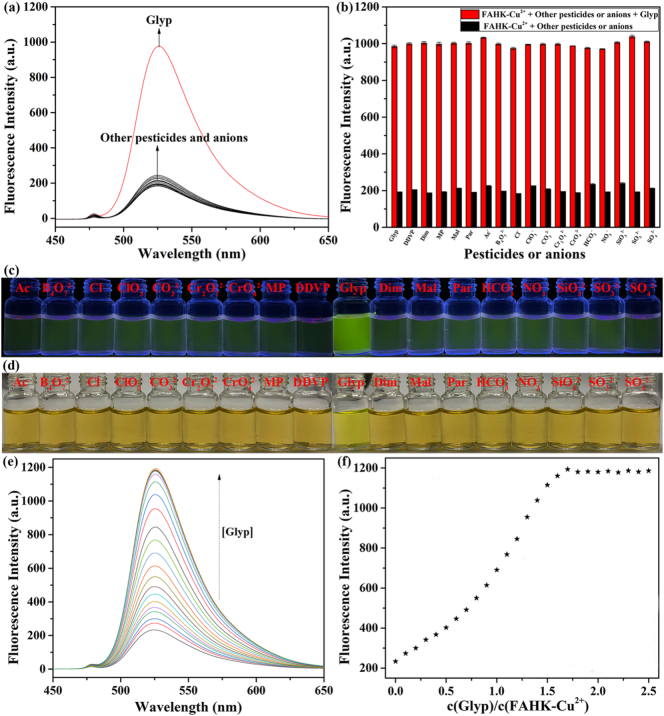
(a) Fluorescence selectivity experiment of **FAHK**-Cu^2+^ ensemble (10 μM) in the presence of various pesticides and anions (10 μM). (b) Fluorescence anti-interference experiment of **FAHK**-Cu^2+^ ensemble (10 μM) with glyphosate (10 μM) in the presence of competitive pesticides and anions (50 μM). Visible color change of **FAHK**-Cu^2+^ ensemble towards pesticides and anions under 365 nm UV light (c) and natural light (d). (e) Fluorescence spectra of **FAHK**-Cu^2+^ ensemble (10 μM) in the presence of different glyphosate concentrations (0–30 μM). (f) Linear relationship between the fluorescence intensity and glyphosate concentration.

The glyphosate (Glyp) molecule contains many functional groups that can form strong coordination bonds with Cu^2+^ to form stable chelate complexes, such as carboxyl, phosphate, and amino groups, etc., so the in situ generated non-fluorescence **FAHK**-Cu^2+^ ensemble can highly selectively detect glyphosate as a fluorescence and colorimetric dual-signals relay response probe. To explore the specific recognition ability of **FAHK**-Cu^2+^ ensemble on glyphosate, the selective recognition properties of **FAHK**-Cu^2+^ ensemble were investigated. The fluorescence intensity of **FAHK**-Cu^2+^ ensemble was greatly enhanced upon addition of glyphosate, while no significant changes were observed in the presence of other pesticides and anions (Fig. 3a). Similarly, the UV–Vis spectra of **FAHK**-Cu^2+^ ensemble with various pesticides and anions were performed. Only glyphosate caused an enhancement of the UV–Vis spectra peak of the **FAHK**-Cu^2+^ ensemble interaction at 495 nm, while the UV-Visintensity of the **FAHK**-Cu^2+^ ensemble did not change significantly after the addition of other pesticides and anions (Fig. S13). Meanwhile, we systematically assessed its response against potential interferents including various pesticides and anions. As observed in Fig. 3b, the fluorescence intensity did not change significantly when other pesticides and anions were added in the presence of glyphosate, indicating that the ability of **FAHK**-Cu^2+^ ensemble for the detection of glyphosate was obvious in the presence of excessive other interference analytes. These results further confirmed **FAHK**-Cu^2+^ ensemble exhibited excellent selectivity and robust interference resistance in detecting glyphosate. Obviously, under 365 nm UV irradiation, **FAHK**-Cu^2+^ ensemble exhibited the typical quenching fluorescence color and only showed a significant recovery when glyphosate was present (Fig. 3c). Similarly, the addition of glyphosate also caused the color to significantly change from dark yellow to greenish-yellow under natural light (Fig. 3d). The data of glyphosate fluorescence titration was completely consistent with the CIE chromaticity coordinates (Fig. S14). Additionally, we investigated the fluorescence titration test to evaluate the recognition capability of **FAHK**-Cu^2+^ ensemble for glyphosate. The addition of glyphosate to **FAHK**-Cu^2+^ ensemble triggered a pronounced fluorescence enhancement (Fig. 3e and Fig. 3f). Based on the 3*σ*/*k* criterion, the LOD for glyphosate was calculated to be 66.3 nM (Fig. S15).

### 3.3 Reversibility, response time and pH effect

**Fig. 4:**
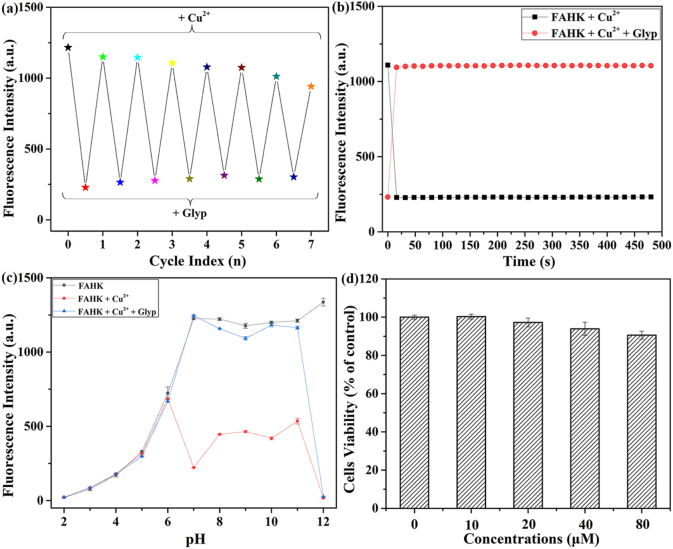
(a) Fluorescence intensity line diagram of **FAHK** (10 μM) with alternately addition of Cu^2+^ (10 μM) and glyphosate (10 μM). (b) The time-dependent fluorescence response for **FAHK** (10 μM) with Cu^2+^ (10 μM) and glyphosate (10 μM). (c) Fluorescence spectra of **FAHK** (10 μM) with Cu^2+^ (10 μM) and glyphosate (10 μM) at different pH values. (d) MTT assay of **FAHK** in living RM1 cells.

To verify the specific fluorescence performance of the probe, we investigated the recycled reversibility experiment of **FAHK** by alternating Cu^2+^ and glyphosate. The fluorescence intensity of **FAHK** was reversibly modulated through alternating additions of Cu^2+^ and glyphosate, with the switching cycle remaining stable for over 7 cycles (Fig. 4a), which demonstrated that **FAHK** exhibited excellent reversible cyclic property for identifying Cu^2+^ and glyphosate. In addition, we perfected the reversible experiments of **FAHK** with Cu^2+^ and glyphosate using colorimetric method (Fig. S16). The color of **FAHK** solution exhibited a significant cyclic change when the alternate addition of Cu^2+^ and glyphosate to the **FAHK** solution under natural light. These results indicated that **FAHK** was a reversible probe that can achieve multiple fluorescence “on-off-on” and colorimetric reversible type repeated detection of Cu^2+^ and glyphosate. On this basis, the reversible titrations experiment of **FAHK** with Cu^2+^ and EDTA was carried out. As shown in Fig. S17, the alternate addition of Cu^2+^ and EDTA to the **FAHK** solution gave rise to a switchable change in the fluorescence intensity and cycle effect was very good with more than 7 times, indicating that **FAHK** can be developed as a reversible fluorescence “on-off-on” probe for Cu^2+^ and EDTA.

Additionally, the time-dependent fluorescence response of **FAHK** to Cu^2+^ and glyphosate was carried out (Fig. 4b). It is worth noting that the emission intensity of **FAHK** decreased sharply within 25 s after Cu^2+^ was added and then remained stable. Subsequently, the fluorescence intensity of **FAHK**-Cu^2+^ ensemble that had already been quenched showed a continuous upward trend after the addition of glyphosate, reaching a plateau approximately 25 s, and the fluorescence intensity basically did not change in the following period. Result of response time suggested that **FAHK** demonstrated an excitingly rapid response and excellent stability during the detection process. Furthermore, the fluorescence intensity of **FAHK** with Cu^2+^ and glyphosate was also performed at different pH (Fig. 4c). Only **FAHK** remained strong fluorescence intensity and good stability under neutral and alkaline conditions (7–12). Upon interaction with Cu^2+^, the fluorescence of **FAHK** was completely quenched within the pH range of 2 to 12. When glyphosate was identified by the **FAHK**-Cu^2+^ complex, a significant increase in fluorescence intensity was observed over the pH range of 7–11. Moreover, the effect of pH on fluorescence intensity of 5-FAM fluorophore with Cu^2+^ and glyphosate was performed within the pH range of 2 to 12 (Fig. S18). 5-FAM fluorophore exhibited strong fluorescence intensity within the range of 7–12. Considering that the pH value range within the human body is between 7.0 and 7.4, 5-FAM fluorophore has great potential for practical application in the field of biology. After the addition of Cu^2+^ and glyphosate, the fluorescence intensity of 5-FAM fluorophore did not show significant changes compared to its only existence, indicating that the tripeptide backbone (Ala-His-Lys-NH_2_) coordinates with Cu^2+^, and the fluorescence intensity of 5-FAM fluorophore did not change due to the addition of Cu^2+^ and glyphosate. Given that the physiological pH range was between 7 and 7.4 in the human body, **FAHK** can be applied to achieve the recognition of Cu^2+^ and glyphosate in biological systems. The fluorescence lifetime decay experiment was carried out and the lifetime of **FAHK** and **FAHK**-Cu^2+^ ensemble were 3.94 ns and 3.85 ns. The fluorescence lifetime was increased to 3.93 ns when glyphosate was added to **FSH**-Cu^2+^ ensemble (Fig. S19 and Table S2).

### 3.4 Bioimaging applications of **FAHK**

**Fig. 5:**
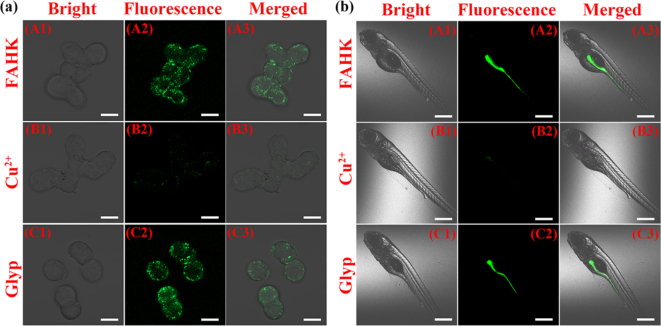
(a) Confocal microscopy images for **FAHK** (10 μM) with Cu^2+^ (10 μM) and glyphosate (10 μM) in living RM1 cells. Scale bars: 10 μm. (b) Confocal microscopy images for **FAHK** (10 μM) with Cu^2+^ (10 μM) and glyphosate (10 μM) in zebrafish larvae. Scale bars: 200 μm.

Taking into account the potential safety of the probe in biological systems, the cytotoxicity of **FAHK** was examined in living RM1 cells using MTT assay. For this assay, primarily the cells were incubated with different concentrations (0, 10, 20, 40 and 80 μM) of **FAHK** for 24 h at 37 °C. Even at a higher concentration (80 μM), the cell viability remained above 94% (Fig. 4d), indicating that **FAHK** demonstrated the low cytotoxicity and favorable biocompatibility, and ensured its further application in biological imaging. Consequently, fluorescence microscopy imaging experiments were performed in living RM1 cells (Fig. 5a). When RM1 cells incubated with **FAHK** (10 μM) for 30 min, strong green fluorescence enhancement was observed (A1-A3). By contrast, RM1 cells incubated with **FAHK** (10 μM) for 30 min and then treated with Cu^2+^ (10 μM) for another 30 min, which showed weak fluorescence in the green channel (B1-B3). Upon adding glyphosate to the cell culture medium containing **FAHK**-Cu^2+^ ensemble for another 30 min, significant green fluorescence was observed again (C1-C3).

Since then, fluorescence imaging of **FAHK** with Cu^2+^ and glyphosate were systematically investigated in zebrafish larvae in order to further verify its potential for biological imaging (Fig. 5b). Strong green signals was detected after the zebrafish larvae were incubated with **FAHK** (10 μM) for 60 min (A1-A3). Notably, the fluorescence of zebrafish larvae was significantly diminished after pretreatment with 10 μM **FAHK** and 10 μM Cu^2+^ for 60 min (B1-B3). And then treatment with glyphosate (10 μM) in above zebrafish larvae and incubating for 60 min, a significant enhancement of fluorescence in green channel was observed (C1-C3). The fluorescence imaging experiments demonstrated that **FAHK** exhibited outstanding detection performance in biological systems and a promising potential for various biological applications.

### 3.5 Measurement in real samples

In order to further demonstrate its practicality in real samples, the spiked recovery experiments were performed in real food samples, including watermelon juice, apple juice, and hami melon juice (Li, Meng, et al., 2025; Zhou et al., 2025). As outlined in Table 1, **FAHK** exhibited remarkable recovery rates of Cu^2+^ ranged from 89.6% to 116.6%, and RSD values of 0.18–2.05% analysis were obtained. In addition, the recovery rates of glyphosate from 84.7% to 111.7% in real food samples, and RSDs lower than 5% (Table 2). Subsequently, the recovery rate tests were conducted in three real water samples in order to further verify the reliability of **FAHK** detection. The recovery rates of Cu^2+^ was calculated to be 89.6%-116.6%, with RSDs less than 2.1% (Table 3). Similarly, the recovery rates for glyphosate ranged from 84.7% to 111.7%, with the RSDs below 5.0% (Table 4). These outcomes results verified that **FAHK** has high accuracy and satisfactory reliability to detecting Cu^2+^ and glyphosate in actual food and water samples. Moreover, **FAHK** was also used to determine Cu^2+^ and glyphosate in two real vegetables samples (lettuce juice and cabbage juice) using the standard recovery experiment. As disclosed in Table S3, the recoveries of Cu^2+^ ranged from 85.2% to 118.8% with the RSD range of 0.51% to 4.05%. Similarly, the recoveries of glyphosate was determined to be in the range from 90.4% to 112.9%, and the RSD was lower than 3.00% (Table S4). Results of experiments proved that **FAHK** can effectively detect Cu^2+^ and glyphosate in real vegetables samples based on satisfactory accuracy and good precision.

**Table 1:** Detection of Cu^2+^ in real food samples.

Sample	Cu^2+^ spiked (μM)	Cu^2+^ found (μM) & % RSD (*n* = 3)	Recovery (%)
Watermelon juice	0	Not detected	
	0.5	0.47 ± 0.36	94.6
	1	0.93 ± 0.49	93.2
	1.5	1.47 ± 0.36	98.2
	2	1.88 ± 0.46	94.1
	2.5	2.23 ± 0.29	89.1
Apple juice	0	Not detected	
	0.5	0.43 ± 1.58	86.5
	1	1.02 ± 1.76	102.1
	1.5	1.75 ± 0.43	116.5
	2	2.34 ± 0.25	117.3
	2.5	2.65 ± 0.12	106.1
Hami melon juice	0	Not detected	
	0.5	0.43 ± 0.31	86.4
	1	0.94 ± 0.42	93.5
	1.5	1.52 ± 0.74	101.4
	2	1.99 ± 1.42	99.4
	2.5	2.44 ± 0.27	97.6

**Table 2:** Detection of Glyp in real food samples.

Sample	Glyp spiked (μM)	Glyp found (μM) & % RSD (n = 3)	Recovery (%)
Watermenlon juice	0	Not detected	
	0.5	0.54 ± 0.54	107.8
	1	1.03 ± 0.74	103.4
	1.5	1.45 ± 0.24	96.7
	2	1.78 ± 0.18	89.2
	2.5	2.39 ± 0.14	95.7
Apple juice	0	Not detected	
	0.5	0.58 ± 1.61	115.3
	1	1.09 ± 0.41	108.5
	1.5	1.39 ± 0.19	92.4
	2	1.79 ± 0.20	89.3
	2.5	2.26 ± 0.15	90.5
Hami melon juice	0	Not detected	
	0.5	0.55 ± 1.15	110.1
	1	1.07 ± 1.42	106.7
	1.5	1.48 ± 0.85	98.5
	2	1.96 ± 1.14	98.0
	2.5	2.28 ± 1.05	91.2

**Table 3:** Detection of Cu^2+^ in real water samples.

Sample	Cu^2+^ spiked (μM)	Cu^2+^ found (μM) & % RSD (n = 3)	Recovery (%)
Lake water	0	Not detected	
	1	1.16 ± 2.05	115.7
	2	1.79 ± 1.46	89.6
	3	3.01 ± 0.66	100.4
Purified water	0	Not detected	
	1	1.12 ± 1.29	111.8
	2	2.26 ± 0.69	112.9
	3	3.50 ± 0.18	116.6
Tap water	0	Not detected	
	1	1.03 ± 1.83	102.5
	2	2.07 ± 0.95	103.5
	3	2.69 ± 0.42	89.8

**Table 4:** Detection of Glyp in real water samples.

Sample	Glyp spiked (μM)	Glyp found (μM) & % RSD (n = 3)	Recovery (%)
Purified water	0	Not detected	
	1	1.12 ± 4.83	111.7
	2	1.97 ± 0.29	98.7
	3	2.76 ± 0.33	91.9
Tap water	0	Not detected	
	1	0.85 ± 0.43	84.7
	2	1.69 ± 1.61	94.1
	3	2.77 ± 1.64	92.2
Lake water	0	Not detected	
	1	0.87 ± 2.13	87.4
	2	1.85 ± 0.14	92.5
	3	3.08 ± 0.32	102.5

### 3.6 Application of test strips

**Fig. 6:**
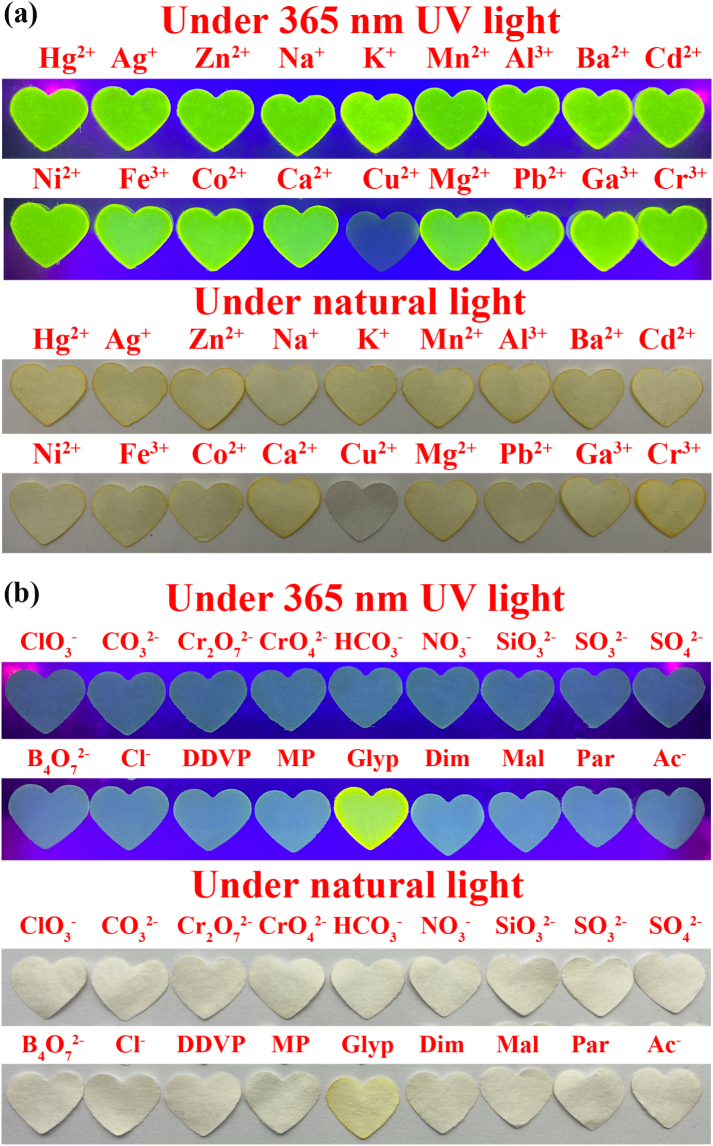
(a) Test strips experiment of **FAHK** with various metal ions under 365 nm UV light and natural light; (b) Test strips experiment of **FAHK**-Cu^2+^ ensemble with various pesticides and anions under 365 nm UV light and natural light.

Moreover, test strips were fabricated using filter papers were soaked by **FAHK** solution. The pre-cut heart-shaped filter paper were respectively immersed in 10 μM **FAHK** solutions and 10 μM **FAHK**-Cu^2+^ ensemble solutions for 30 min and then dried. When various metal ion solutions were absorbed onto the test strips, only the test strip soaked in Cu^2+^ exhibited fluorescence quenching under UV light at 365 nm, as well as changed from beige color to olive gray under natural light (Fig. 6a). Similarly, only adding glyphosate to the test strip containing **FAHK**-Cu^2+^ ensemble significantly restored the color of the quenched test strip, while the presence of other interfering pesticides and anions caused negligible changes (Fig. 6b). The above experimental results further confirmed that the developed test strips exhibited excellent sensing selectivity during the visual detection process with the merits of high specificity, fast response speed and visual signal output.

### 3.7 Logic gate construction

**Fig. 7:**
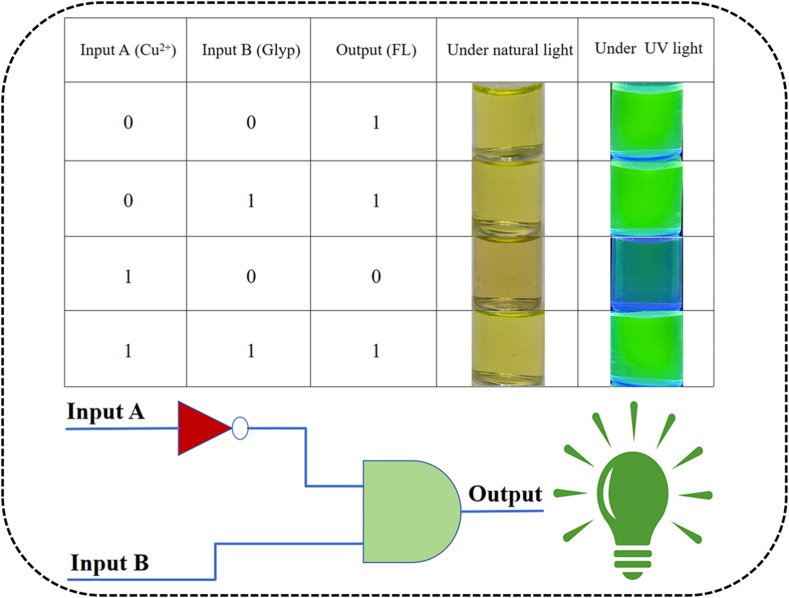
INHIBIT molecular logic gate representation of **FAHK** using truth table and gate notation.

Based on the significant “on-off-on” emission characteristic and colorimetric color change of **FAHK**, the INHIBIT logic gate was designed. In this regard, Cu^2+^ and glyphosate were applied as input A and input B, which defined that their absence as “0” and their presence as “1”. The fluorescence intensity and the solution color were set as the output. When the fluorescence intensity increases, the output signal was represented as 1 (“on” state), while it is represented as 0 (“off” state) when the fluorescence intensity was quenched. As shown in Fig. 7, the fluorescence intensity of **FAHK** was extremely weak and the output was 0 when only Cu^2+^ (1,0) was input. The strong fluorescence intensity was observed in the absence (0,0), only glyphosate (0,1), and both inputs (1,1). In addition, the color of the solution appeared as a bright green under 365 nm UV light, as well as all turned greenish-yellow under natural light. As an outstanding dual-signals probe, **FAHK** demonstrated great potential in the construction of logic gate detection.

### 3.8 Smartphone-based visual detection

**Fig. 8:**
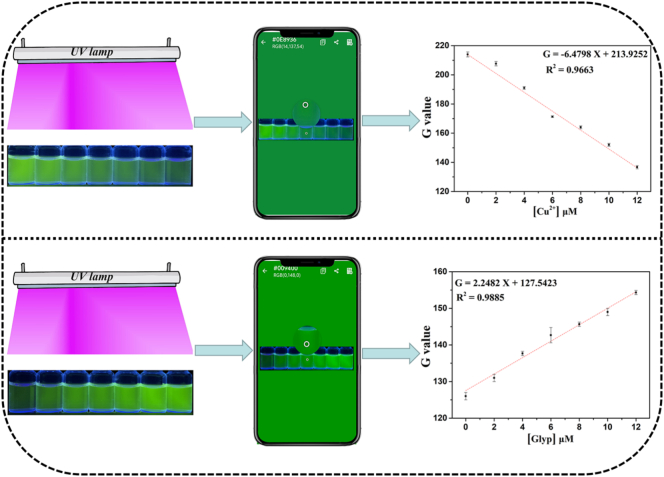
Schematic diagram of the smartphone-assisted sensing platform for the quantitative detection of Cu^2+^ and glyphosate.

With the rapid development of detection technology, the construction of simple and convenient visual detection devices has become an inevitable new trend. To achieve practical and quantitative monitoring of Cu^2+^ and glyphosate, the smartphone-based color recognition *App* was introduced as a portable tool. Using the smartphone to capture the fluorescence images of the probe solutions, and through the color recognition application, the RGB (red, green, blue) color values was extracted from the images (Fig. 8, Table S5 and Table S6). By increasing the amount of Cu^2+^ in range of 0–12 μM, the fluorescence color of **FAHK** gradually changed from light green to dark green under the 365 nm UV lamp. The G value exhibited strong linear correlations with the Cu^2+^ concentrations, followed the equation G = −6.4798 X + 213.9252 (R^2^ = 0.9663). Similarly, **FAHK**-Cu^2+^ ensemble's color gradually intensifying from dark green to light green with an increase in glyphosate. Subsequently, G value achieved a wider linear range for glyphosate concentration of 0–12 μM (G = 2.2482 X + 127.5423, R^2^ = 0.9885). The LODs of smartphone detection for Cu^2+^ and glyphosate were determined to be 131.4 nM and 87.3 nM using the smartphone assisted RGB method. The portable sensing platform based on smartphones has unique advantages such as portability, rapid response and high sensitivity. It enables rapid and visual detection of Cu^2+^ and glyphosate, thereby eliminating the reliance on expensive laboratory equipment during the detection process.

### 3.9 Fluorescence imaging of food surfaces

**Fig. 9:**
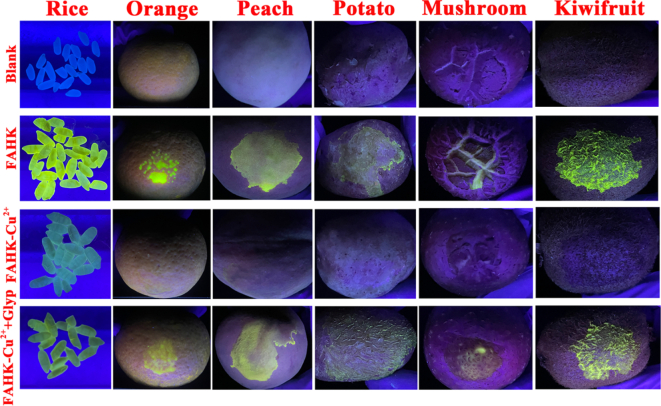
Fluorescence color images of **FAHK** towards Cu^2+^ and glyphosate residues on various food surfaces under 365 nm UV light.

In order to further expand the potential application fields of the probe, **FAHK** were used to detect Cu^2+^ and glyphosate on six food surfaces, including rice, orange, peach, potato, mushroom, and kiwifruit (Fig. 9). Noteworthy, the food surfaces that were not sprayed with **FAHK** solution did not show any fluorescent color under 365 nm UV light. The bright yellow fluorescence was observed when the **FAHK** solution was sprayed on the surface of the food and then left to dry naturally at room temperature. However, the treated surface of the sample after sprayed with **FAHK**-Cu^2+^ ensemble solution exhibited a distinct fluorescence quenching change with naked eyes. Further, when six food surfaces sprayed with **FAHK**-Cu^2+^ ensemble solution and then sprayed by glyphosate, strong yellow fluorescence on food surfaces were also significantly enhanced. The experimental results clearly demonstrated that **FAHK** exhibited excellent advantages in the detection of Cu^2+^ and glyphosate on various food surfaces, which also provided a very feasible method for quickly and visually identifying pesticide residues in food.

### 3.10 Analysis of glyphosate residues in soil sample

**Fig. 10:**
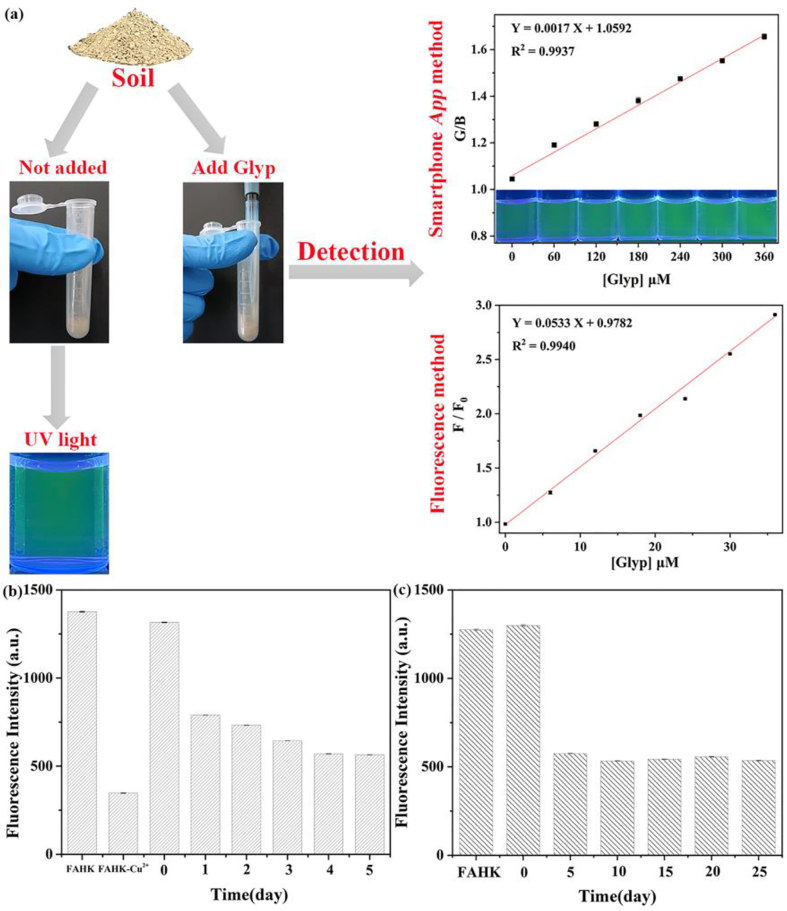
(a) Schematic diagram of **FAHK**-Cu^2+^ ensemble for detecting glyphosate in soil samples using two methods. Fluorescence intensity of **FAHK**-Cu^2+^ ensemble with glyphosate residues in soil samples within 0–5 days (b) and 0–25 days (c).

Glyphosate has high environmental stability and remains highly active and resistant to degradation in the soil after application, so it is urgently necessary to further assess the residues of glyphosate in the soil sample using **FAHK**-Cu^2+^ ensemble. The dark green color of the solution was observed without glyphosate (Fig. 10a). With the increase in the concentrations of glyphosate, the color of **FAHK**-Cu^2+^ ensemble solution changes from blue-green to bright green under 365 nm UV light, a significant linear relationship was noted between G/B and glyphosate, with the corresponding linear equation being G/B = 0.0017× + 1.0592 (R^2^ = 0.9937). Similarly, *F*/*F*_*0*_ demonstrated excellent linearity with glyphosate concentration (*F*/*F*_*0*_ = 0.0533× + 0.9782, R^2^ = 0.9940). Based on the cost-effective, easy-to-operate and highly sensitive fluorescence detection method and the portable sensing technology of smartphone, we achieved efficient and visual detection of glyphosate residues in soil sample. In addition, we determined the degradation of glyphosate in soil samples using **FAHK**-Cu^2+^ ensemble. As depicted in Fig. 10b, the fluorescence intensity gradually decreased within 5 days, indicating that the concentration of glyphosate in the soil sample showed a downward trend over time, which might be due to the degradation of glyphosate by microorganisms and minerals in the soil sample. However, after a period of more than 5 days, the concentration of glyphosate in the soil sample no longer decreased, indicating that the degradation of glyphosate by microorganisms and minerals was limited (Fig. 10c). This study not only provided a new strategy for highly sensitive and portable detection of glyphosate residues in soil sample, but also has potential application value in the field of food safety screening.

## 4 Conclusion

This study successfully designed and synthesized a new dual-signals peptide-based probe **FAHK** through 5-carboxy fluorescein (5-FAM) fluorophore coupled tripeptide backbone (Ala-His-Lys-NH_2_). **FAHK** exhibited high selectivity, excellent sensitivity and extremely low interference effect in the continuous detection of Cu^2+^ and glyphosate. **FAHK** achieved the LODs of 49.7 nM for Cu^2+^ and 66.3 nM for glyphosate, respectively. Additionally, **FAHK** offered a highly promising tool for the quantitative analysis of Cu^2+^ and glyphosate in food samples, as well as was successfully applied in fluorescence imaging studies in biological systems. Besides, **FAHK** loaded test strips were successfully prepared for fluorometric and colorimetric detection of Cu^2+^ and glyphosate. Interestingly, we successfully fabricated an INHIBIT molecular logic gate by taking advantage of the fluorescence “on-off-on” response characteristic of **FAHK**. Notably, a smartphone-based platform the semi-quantitative analysis was successfully developed without the need for complicated device. Additionally, **FAHK** was further employed to image Cu^2+^ and glyphosate residues on surfaces of food samples under 365 nm UV light. More importantly, **FAHK** was successfully applied to monitor the degradation of glyphosate in soil samples with satisfactory results. **FAHK** provided a highly sensitive and rapid visual analysis method for monitoring Cu^2+^ and glyphosate in real food samples, environment and biological imaging. We believe that **FAHK** will offer great potential application prospects in food testing and biological imaging fields.

## CRediT authorship contribution statement

**Yi Ren:** Writing – original draft, Methodology, Formal analysis, Data curation, Conceptualization. **Mengying Jia:** Formal analysis, Data curation. **Shiyi Xiong:** Software. **Yong An:** Writing – review & editing, Software, Funding acquisition. **Xiupei Yang:** Funding acquisition, Formal analysis. **Peng Wang:** Writing – review & editing, Project administration, Funding acquisition, Formal analysis, Data curation, Conceptualization.

## Declaration of competing interest

The authors declare that they have no known competing financial interests or personal relationships that could have appeared to influence the work reported in this paper.

## Data Availability

No data was used for the research described in the article.

## Appendix A Supplementary data

Supplementary material Image 7
