# Supplementary material for: A dual-signals peptide-based probe for fluorometric and colorimetric detection of copper(II) ions and glyphosate in multiple food samples and biological system
Source: Food Chem X. 2026 Mar 21;35:103776. doi: 10.1016/j.fochx.2026.103776 (PMC13050119; doi:10.1016/j.fochx.2026.103776)
Supplement: Supplementary file 1 — Supplementary material [file mmc1.docx]

**Supplementary Material**

**A dual-signals peptide-based probe for fluorometric and colorimetric detection of copper(Ⅱ) ions and glyphosate in multiple food samples and biological system**

*Yi Ren ^a^, Mengying Jia ^a^, Shiyi Xiong ^a^, Yong An ^b^*, Xiupei Yang ^a^*, Peng Wang ^a^**

*^a^* *Precise Synthesis and Function Development Key Laboratory of Sichuan Province, College of Chemistry and Chemical Engineering, China West Normal University, Shida Road 1#, Nanchong 637009, PR. China.*

*^b^ The First School of Clinical Medicine, Gansu University of Chinese Medicine, Lanzhou, Gansu 730030, PR. China.*

** Corresponding Authors*

*Email address: wangpchem17@163.com (P. Wang), smcay123@163.com (Y. An), xiupeiyang@163.com (X. Yang).*

**HPLC chromatogram of probe FAHK**

Sample: **FAHK**

Column: 4.6_*_150 mm, kromasil C18-5

Solvent A: 0.1% Trifluoroacetic acid in 100% Acetonitrile

Solvent B: 0.1% Trifluoroacetic acid in 100% Water

Gradient: Time A B

0.01 min 5% 95%

25.0 min 70% 30%

30.0 min 90% 10%

Flow rate: 1.0 ml/min

Wavelength: 214 nm

Volume: 20 μL

**
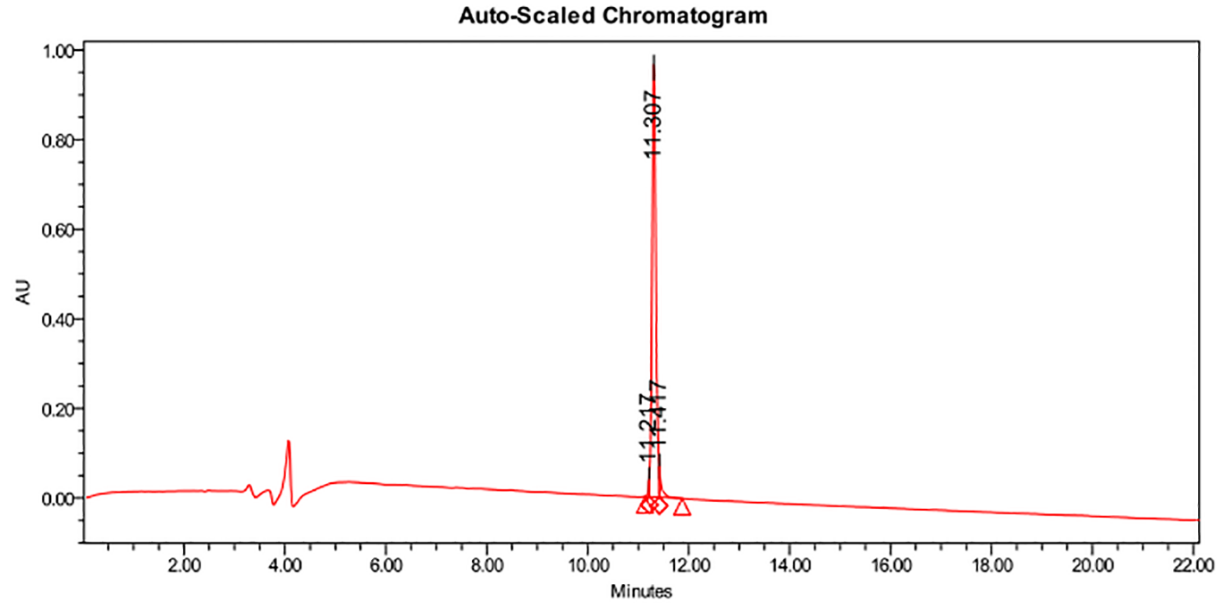
**

**Fig.** **S1.** HPLC chromatogram of probe **FAHK**.

**Table S1.** HPLC data of probe **FAHK**

| Rank | RT | Area | Height | % Area |
| --- | --- | --- | --- | --- |
| 1 | 11.217 | 44996 | 37026 | 0.81 |
| 2 | 11.307 | 5274075 | 968237 | 95.43 |
| 3 | 11.417 | 207651 | 68282 | 3.76 |

**MS Analysis data**

Sample: **FAHK**

Expected MS: 711.70

Observed MS: 712.61

Buffer: Second distilled water (ddH_2_O)


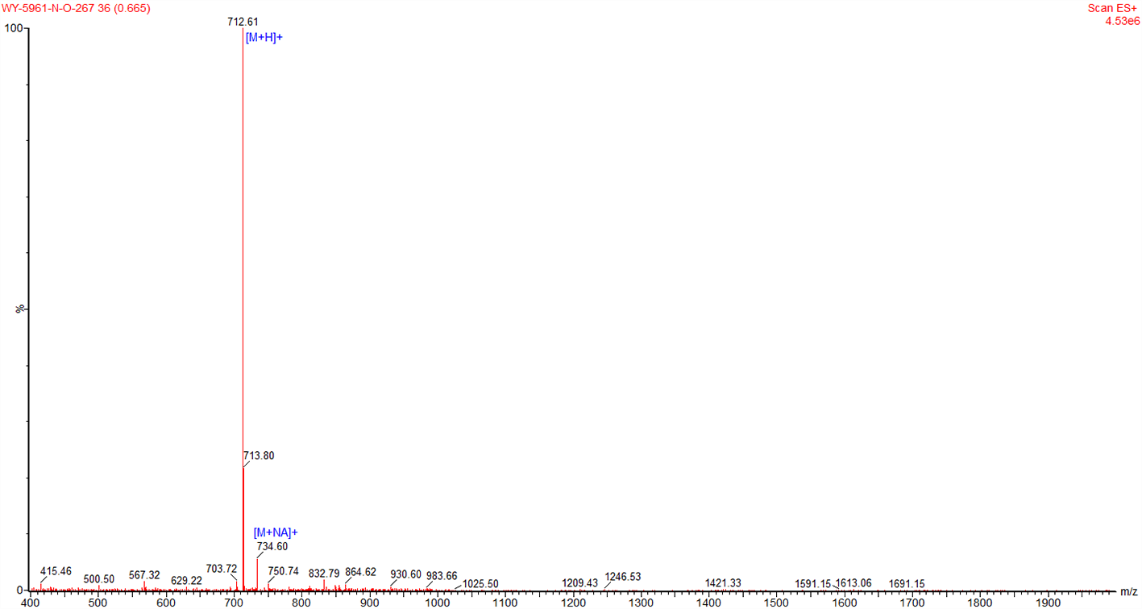


**Fig. S2.** ESI-MS spectrum of probe **FAHK**.

^1^H NMR spectrum of probe FAHK


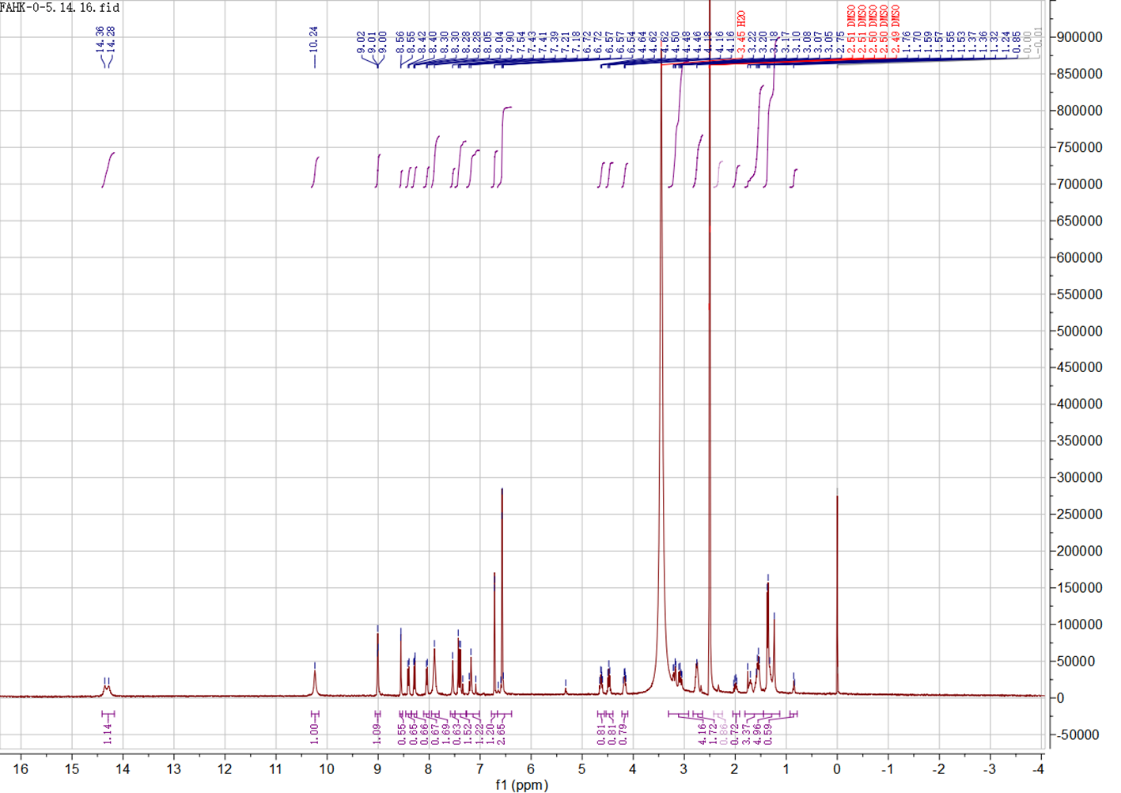


**Fig. S3.** ^1^H NMR spectrum of **FAHK** in DMSO-*d_6_*.

**UV-vis response of FAHK towards Cu^2+^**

**^
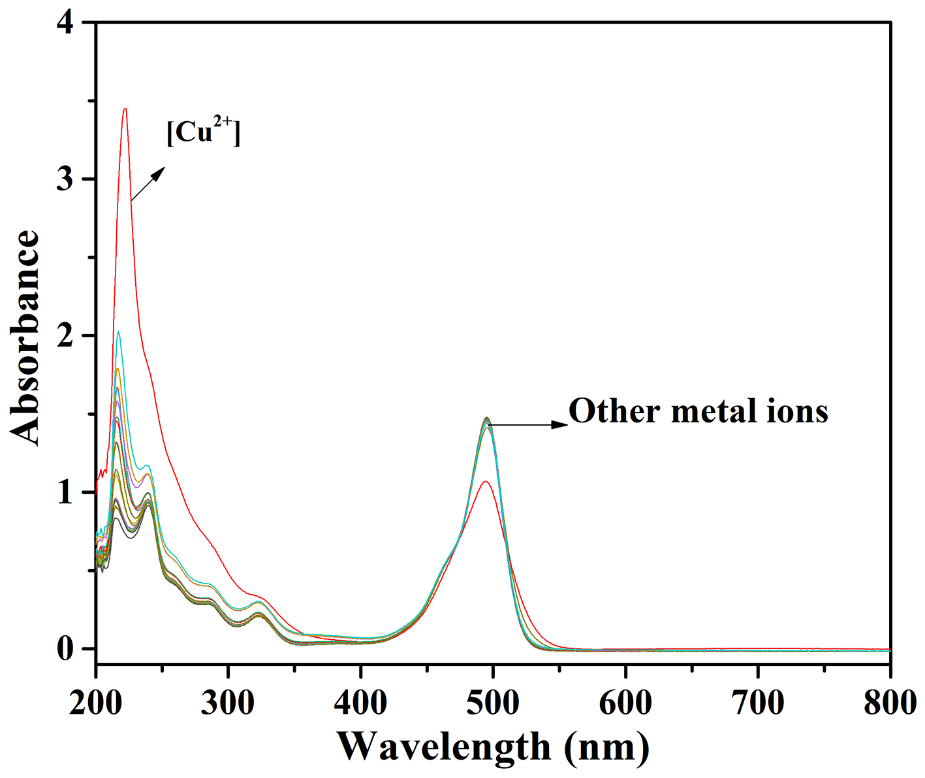
^**

**Fig. S4**. UV-vis spectra of **FAHK** (10 μM) in presence of various metal ions (10 μM) in HEPES buffer (10 mM, pH = 7.4) solutions.

**CIE diagram of FAHK with Cu^2+^**


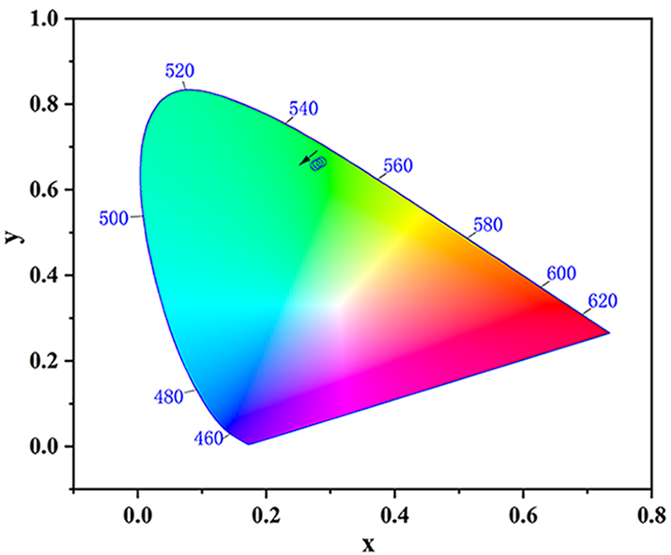


**Fig. S5.** CIE diagram of **FAHK** with different concentrations of Cu^2+^.

**UV-vis absorption of FAHK with Cu^2+^**

**
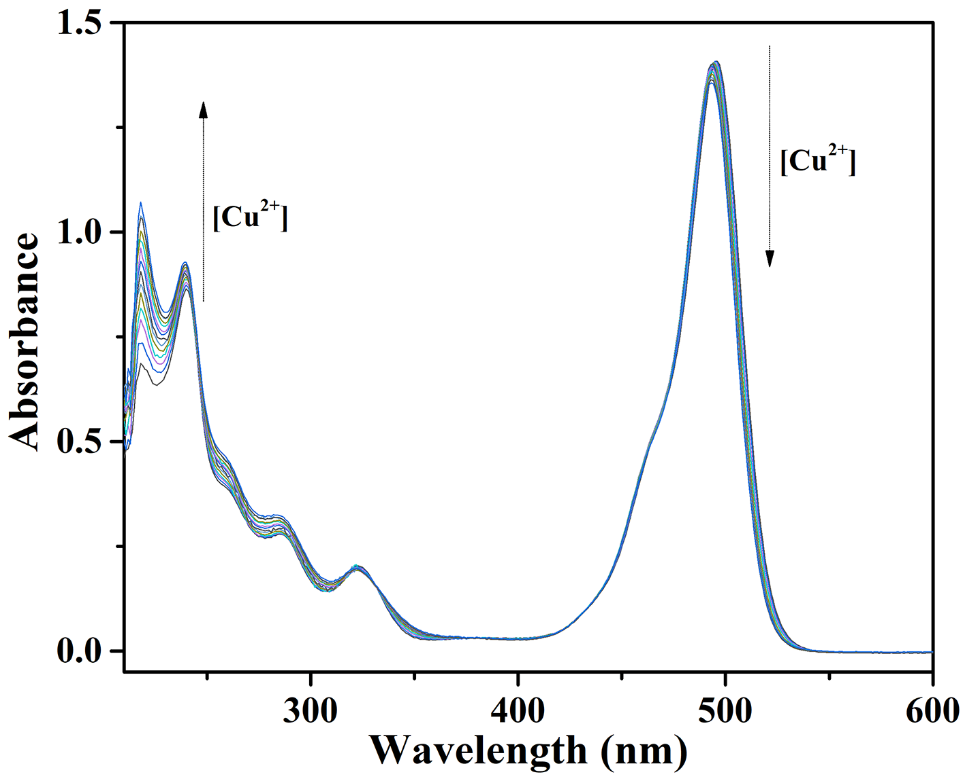
**

**Fig. S6.** UV-vis spectra of **FAHK** (10 μM) upon addition of Cu^2+^ (0-1 equiv.) in HEPES buffer solutions (10 mM, pH 7.4).

**Job’s plot of FAHK with Cu^2+^ ions**

**
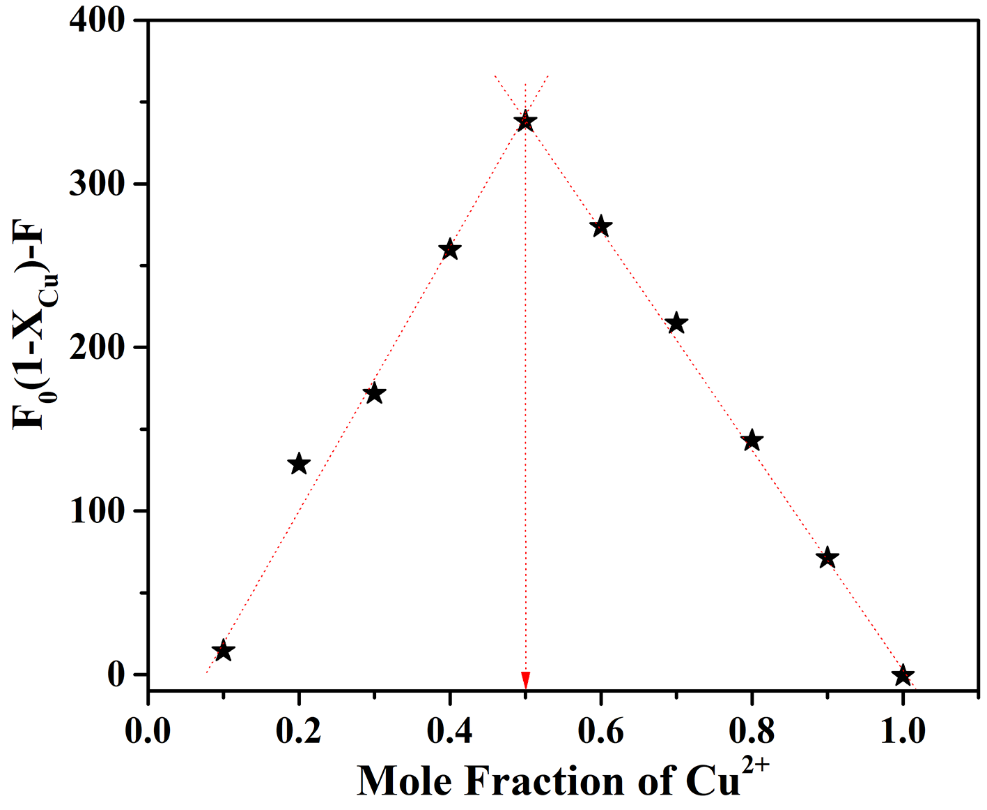
**

**Fig. S7.** Job’s plot for determining the stoichiometry of probe **FAHK** with Cu^2+^ ions.

**ESI-HRMS analysis of FAHK with Cu^2+^ ions**

**^
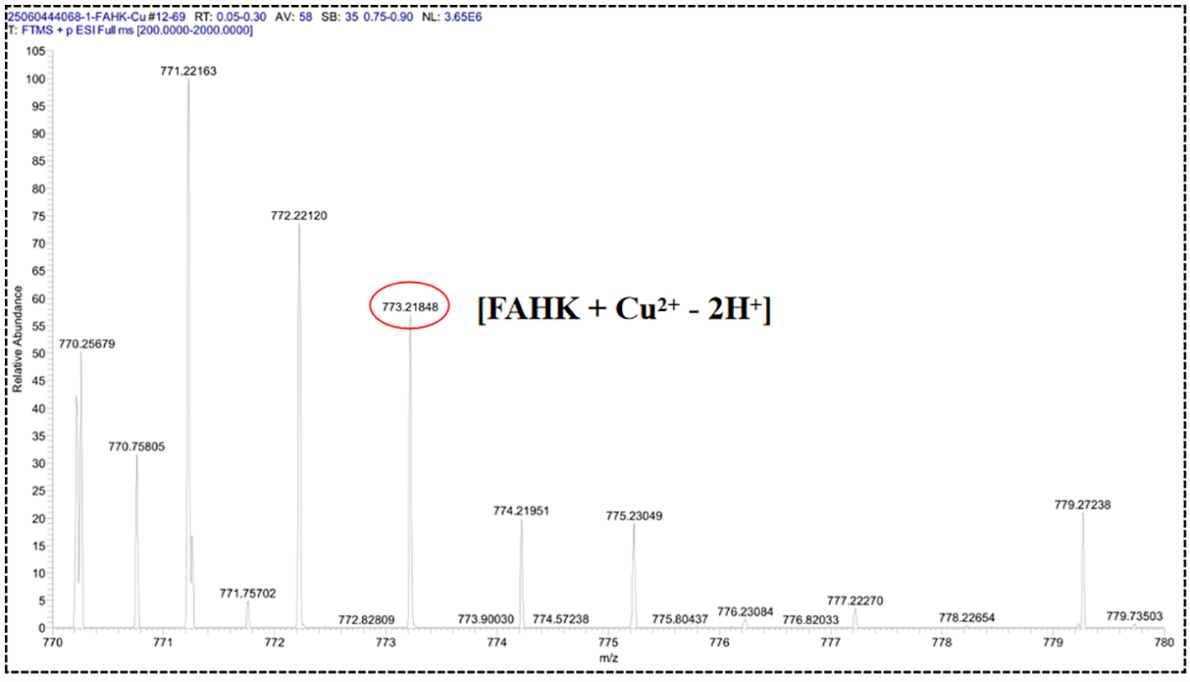
^**

**Fig. S8.** ESI**-**HRMS of probe **FAHK** (100.0 μM) with Cu^2+^ in HEPES buffer (10.0 mM, pH 7.4) solutions.

**The ^1^H NMR spectra analysis of FAHK to Cu^2+^ ions**

**
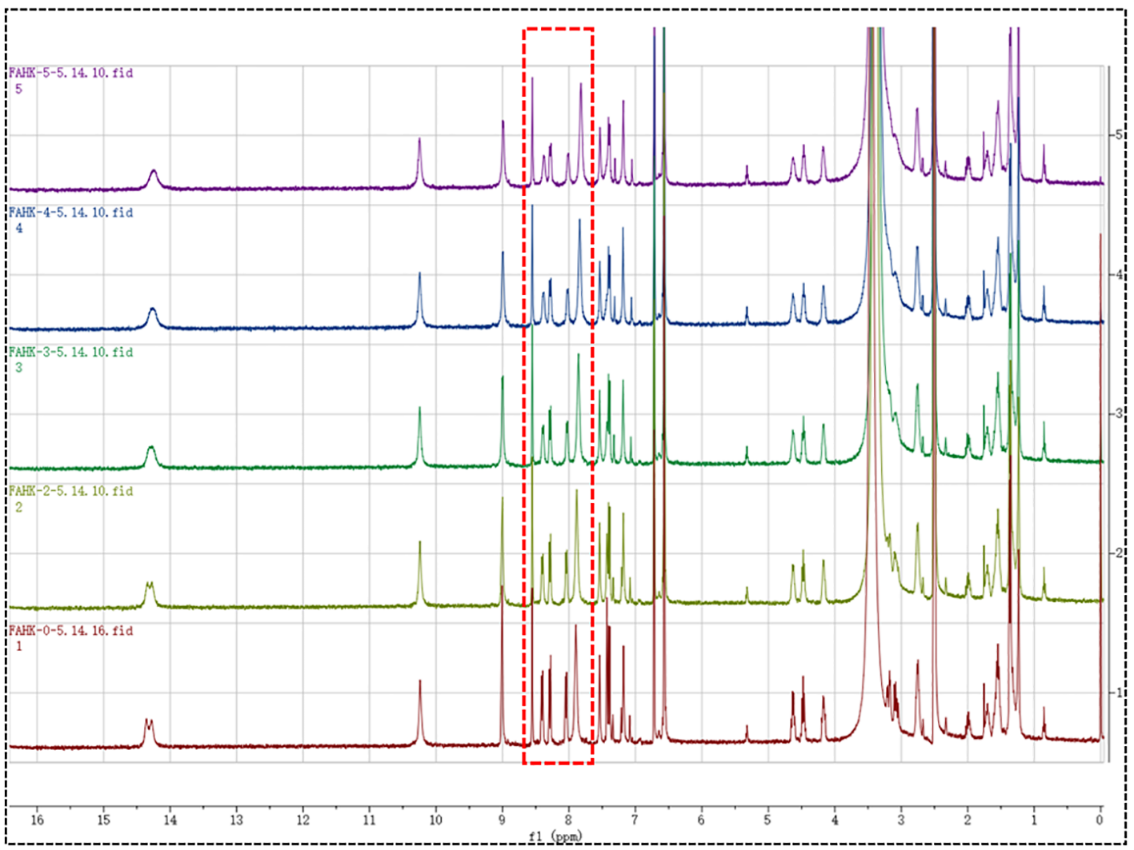
**

**Fig. S9**. ^1^H NMR spectra of **FAHK** in presence of Cu^2+^ (0-0.8 equiv.) in DMSO-*d_6_*.

**FTIR spectra analysis of FAHK to Cu^2+^ ions**

**
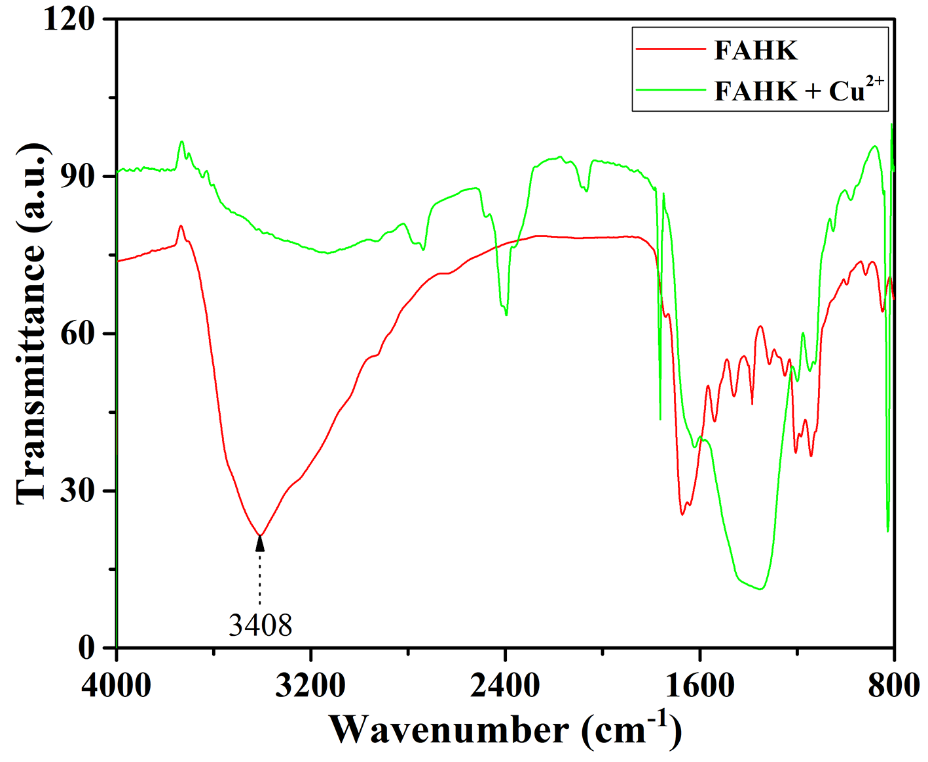
**

**Fig. S10.** FTIR spectra of **FAHK** in the absence and presence of Cu^2+^ ions.

**The binding constant of FAHK and Cu^2+^ ions**

The association constants for 1:1 complex were calculated based on the titration curve of the chemosensors with metal ions. The binding constant was calculated from the emission intensity-titration curves according to the equation:

where *F_0_* is the emission intensity of **FAHK**, *F* is the emission intensity of **FAHK** upon the addition of different amount of Cu^2+^, *f* is the fraction of the initial fluorescence which is accessible to **FAHK**, [*M*] is the concentration of Cu^2+^. The association constant values *K_s_* is given by the ratio intercept/slope.


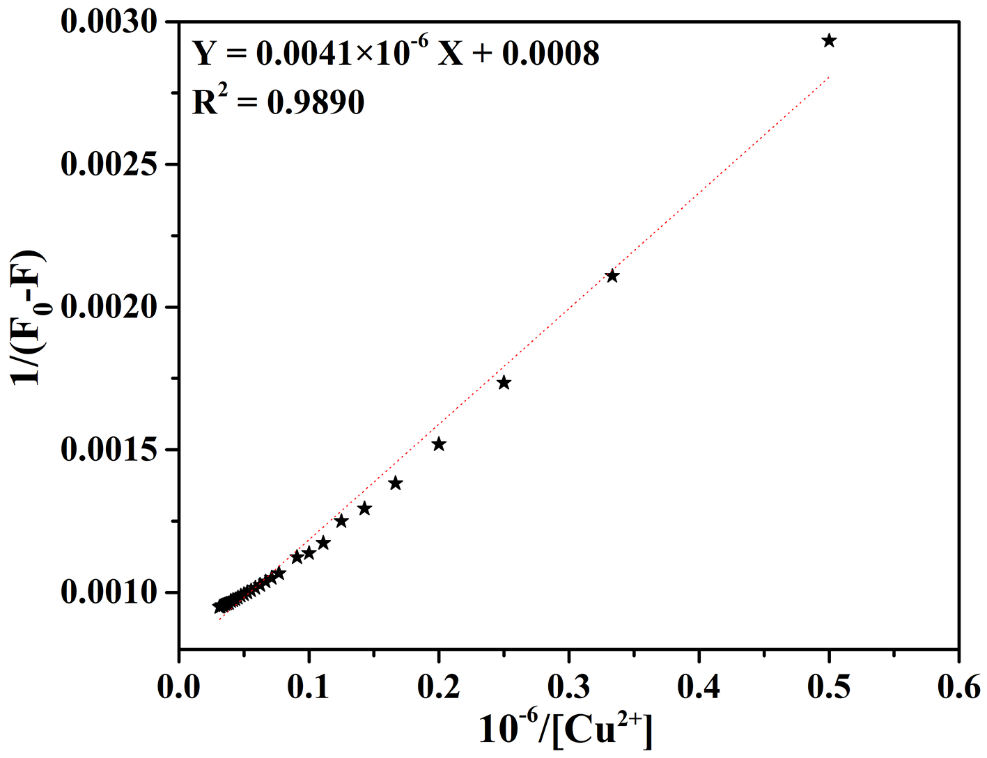


**Fig. S11.** Fitting of fluorescence titration curve of probe **FAHK** with Cu^2+^ in HEPES buffer solutions (10.0 mM, pH 7.4). The binding constant of **FAHK**-Cu^2+^ ensemble was 1.9×10^5^ M^-1^ (R^2^ = 0.9890).

**The detection limit for Cu^2+^**

The limit of detection (LOD) was calculated based on the fluorescence titration of low concentration Cu^2+^. The fluorescence emission intensity of **FAHK** without Cu^2+^ was measured 10 times, and the standard deviation of the blank measurements was determined. A good linear curve relationship between the fluorescence intensity and the Cu^2+^ concentrations was obtained in the low concentrations (0-6.0 μM) range (R^2^ = 0.9911). The LOD was then calculated as 3*σ*/*k*, where *σ* is the standard deviation of the blank measurements, and *k* is the gradient of the fluorescence intensity versus Cu^2+^ concentration.


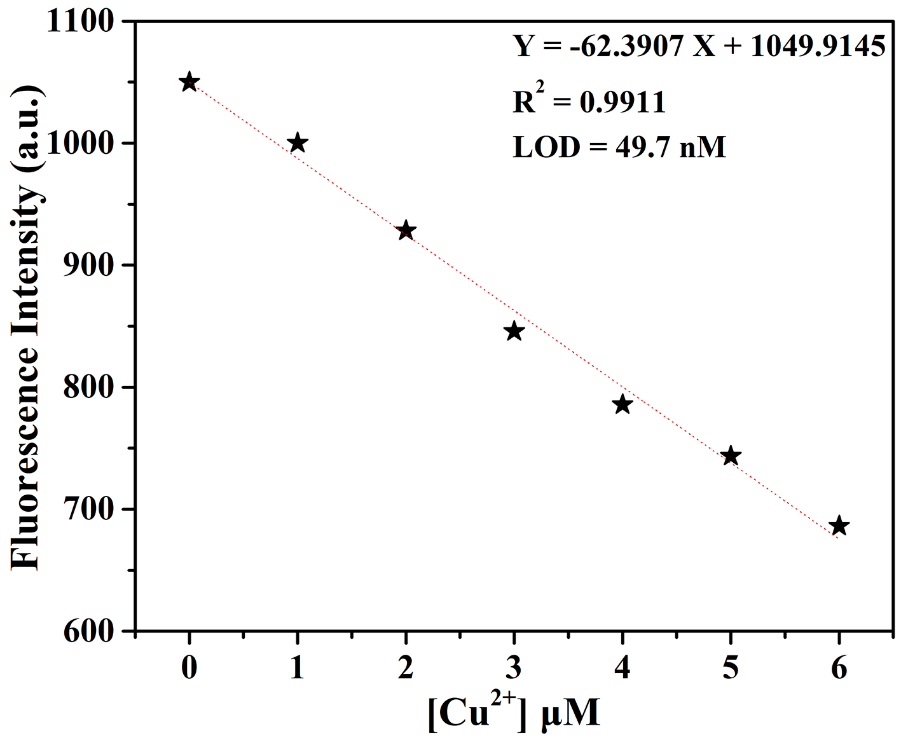


**Fig. S12.** Fluorescence intensity of probe **FAHK** (10.0 μM) with gradient concentration of Cu^2+^ (0-6.0 μM) were added in HEPES buffer solutions (10.0 mM, pH 7.4). The lowest detection limit of Cu^2+^ was 49.7 nM.

**UV-vis response of FAHK-Cu^2+^ ensemble towards glyphosate**

**
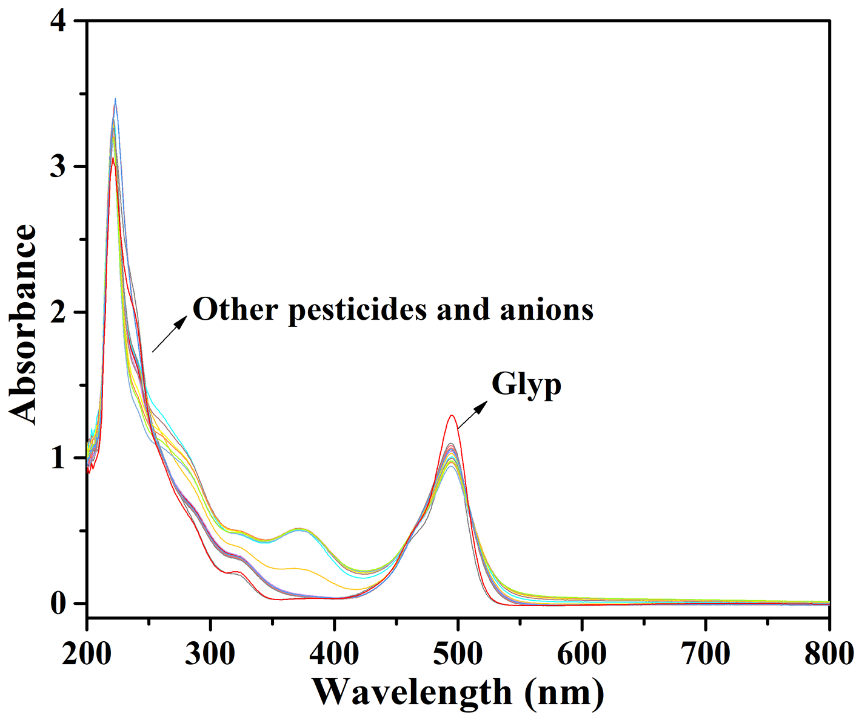
**

**Fig. S13**. UV-vis spectra of the **FAHK**-Cu^2+^ ensemble (10 μM) in presence of various pesticides and anions (10 μM) in HEPES buffer (10 mM, pH = 7.4) solutions.

**CIE diagram of FAHK-Cu^2+^ ensemble with glyphosate**


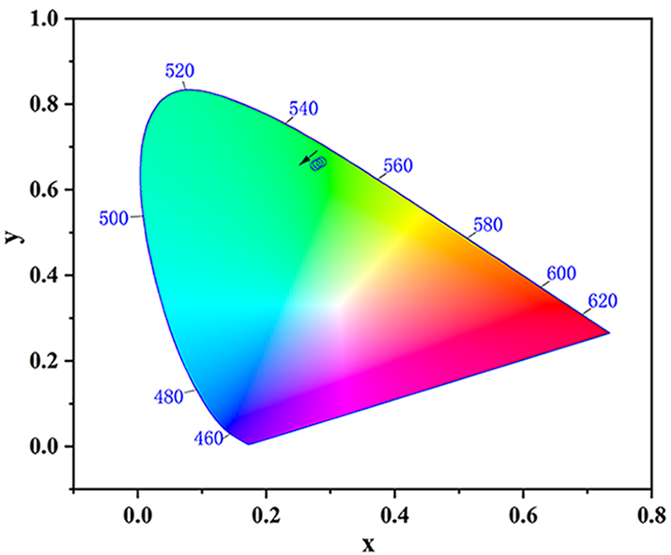


**Fig. S14.** CIE diagram of **FAHK-**Cu^2+^ ensemble with different concentrations of glyphosate.

**The detection limit for glyphosate**

The limit of detection (LOD) was calculated based on the fluorescence titration of low concentration glyphosate. The fluorescence emission intensity of **FAHK-**Cu^2+^ ensemble without glyphosate was measured 10 times, and the standard deviation of the blank measurements was determined. A good linear curve relationship between the fluorescence intensity of **FAHK-**Cu^2+^ ensemble and the glyphosate concentrations was obtained in the low concentrations (0-7.5 μM) range (R^2^ = 0.9942). The LOD was then calculated as 3*σ*/*k*, where *σ* is the standard deviation of the blank measurements, and *k* is the slope of the fluorescence intensity versus glyphosate concentration.


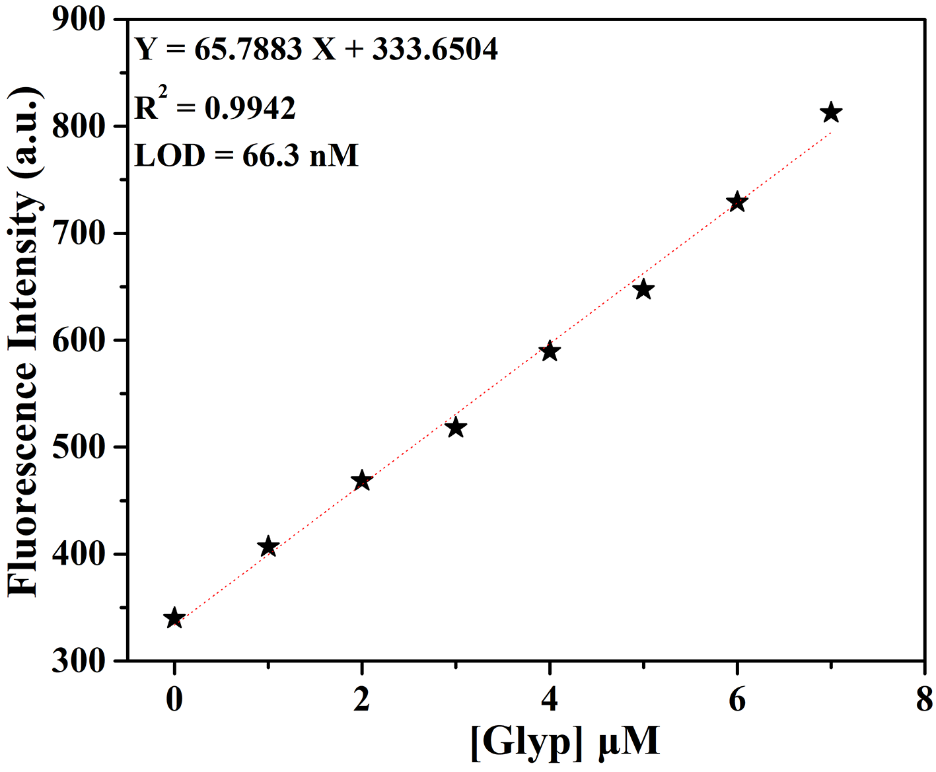


**Fig. S15.** Fluorescence intensity of **FAHK**-Cu^2+^ ensemble (10.0 μM) with gradient concentration of S^2−^ (0-7.5 μM) were added in HEPES buffer solutions (10.0 mM, pH 7.4). The lowest detection limit of glyphosate was 66.3 nM.

**Colorimetric reversible of FAHK with Cu^2+^ and glyphosate**

**
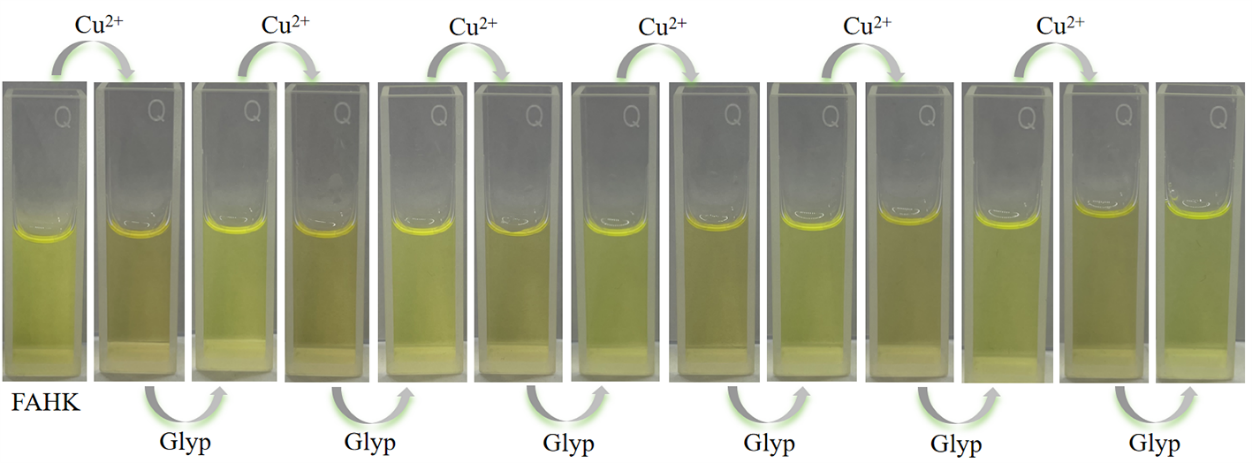
**

**Fig. S16.** Colorimetric reversible of **FAHK** (10 μM) by alternately adding 10 μM Cu^2+^ and 10 μM glyphosate in HEPES buffer (10 mM, pH = 7.4) solutions.

**Reversibility experiment of FAHK with Cu^2+^ and EDTA**

**
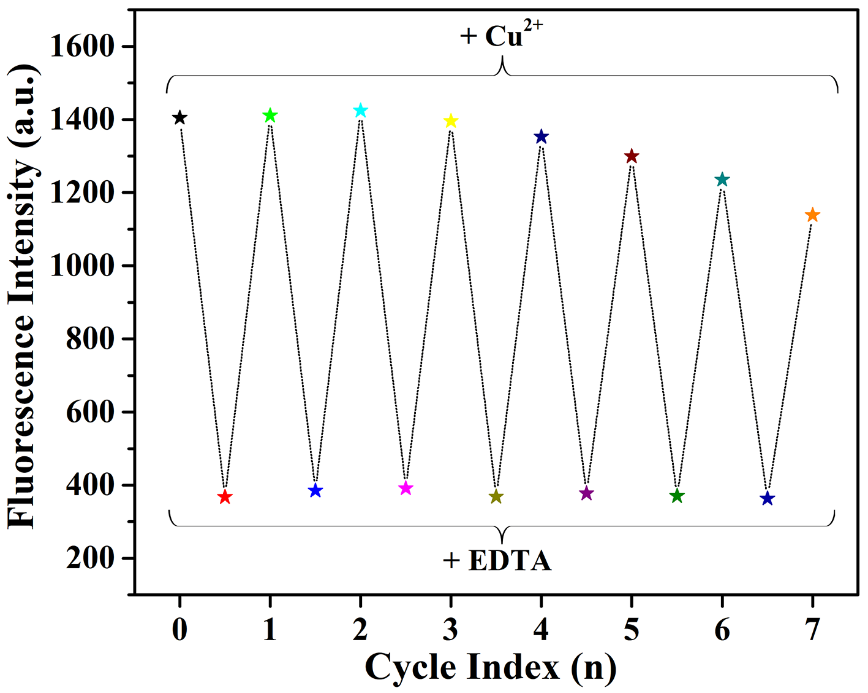
**

**Fig. S17**. Fluorescence emission spectra of **FAHK** (10 μM) by alternately adding 10 μM Cu^2+^ and 10 μM EDTA in HEPES buffer (10 mM, pH = 7.4) solutions.

**pH effect of 5-FAM fluorophore with Cu^2+^ and glyphosate**

**
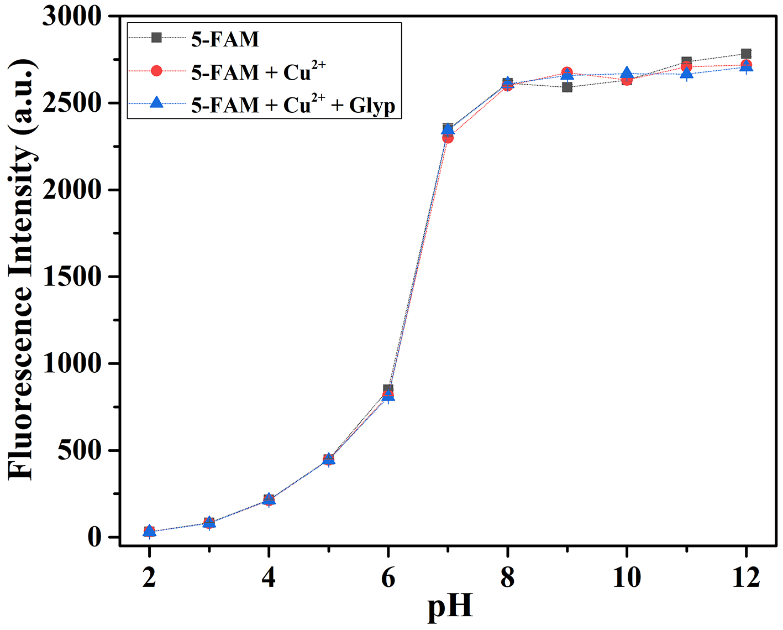
**

**Fig. S18**. The fluorescence intensity of 5-FAM fluorophore in the absence and presence of Cu^2+^ and glyphosate under different pH values (2-12).

**Fluorescence lifetime of FAHK with Cu^2+^ and glyphosate**

**
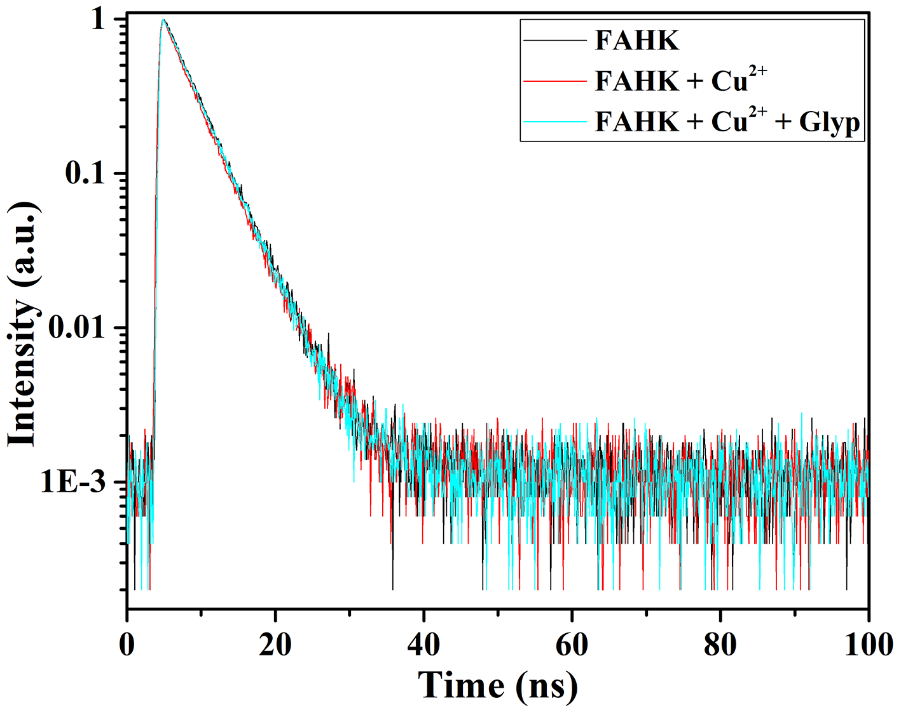
**

**Fig. S19.** Fluorescence lifetime decay profile of **FAHK** (10.0 μM) with Cu^2+^ (1.0 equiv.) and glyphosate (1.0 equiv.).

**Table S2.** Fluorescence lifetime decay data

|  | **τ_1_/ns (α₁/%)** | **tave/ns** |
| --- | --- | --- |
| **FAHK** | 3.94 (100) | 3.94 |
| **FAHK** + Cu^2+^ | 3.85 (100) | 3.85 |
| **FAHK** + Cu^2+^ + Glyp | 3.93 (100) | 3.93 |

**Table S3**.

Determination of Cu^2+^ in real vegetables samples

| Sample | Cu^2+^ spiked (μM) | Cu^2+^ found (μM) & % RSD (n = 3) | Recovery (%) |
| --- | --- | --- | --- |
| Lettuce juice  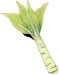 | 0 | Not detected |  |
|  | 1 | 0.87 ± 7.05 | 87.0 |
|  | 2 | 2.38 ± 3.34 | 118.8 |
|  | 3 | 3.45 ± 2.84 | 114.8 |
| Cabbage juice  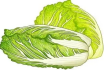 | 0 | Not detected |  |
|  | 1 | 0.85 ± 4.65 | 85.2 |
|  | 2 | 1.71 ± 1.21 | 85.4 |
|  | 3 | 3.03 ± 0.51 | 101.1 |

**Table S4**.

Determination of glyphosate in real vegetables samples

| Sample | Glyp spiked (μM) | Glyp found (μM) & % RSD (n = 3) | Recovery (%) |
| --- | --- | --- | --- |
| Lettuce juice  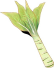 | 0 | Not detected |  |
|  | 0.5 | 0.53 ± 1.44 | 106.9 |
|  | 1 | 1.04 ± 2.70 | 103.6 |
|  | 1.5 | 1.68 ± 1.34 | 112.3 |
| Cabbage juice  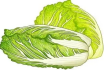 | 0 | Not detected |  |
|  | 0.5 | 0.56 ± 0.41 | 112.9 |
|  | 1 | 0.90 ± 1.42 | 90.4 |
|  | 1.5 | 1.21 ± 0.36 | 80.7 |

**Table S5**

Visual inspection of Cu^2+^ through the color parameters obtained using a smartphone application

| c(Cu^2+^)  μM | R value | | | G value | | | B value | | | G | RSD  (n=3) |
| --- | --- | --- | --- | --- | --- | --- | --- | --- | --- | --- | --- |
|  | 1 | 2 | 3 | 1 | 2 | 3 | 1 | 2 | 3 |  |  |
| 0 | 56 | 58 | 62 | 213 | 213 | 216 | 22 | 49 | 40 | 214 | 1.73 |
| 2 | 52 | 56 | 53 | 206 | 208 | 209 | 5 | 7 | 8 | 207.67 | 1.53 |
| 4 | 43 | 31 | 33 | 192 | 190 | 191 | 46 | 49 | 50 | 191 | 1.00 |
| 6 | 26 | 28 | 26 | 172 | 171 | 171 | 67 | 73 | 69 | 171.33 | 0.58 |
| 8 | 26 | 25 | 23 | 165 | 163 | 164 | 87 | 94 | 85 | 164 | 1.00 |
| 10 | 24 | 29 | 26 | 151 | 153 | 152 | 82 | 84 | 83 | 152 | 1.00 |
| 12 | 34 | 31 | 30 | 138 | 136 | 136 | 94 | 92 | 91 | 136.67 | 1.15 |

**Table S6**

Visual inspection of glyphosate through the color parameters obtained using a smartphone application

| c(Glyp)  μM | R value | | | G value | | | B value | | | G | RSD  (n=3) |
| --- | --- | --- | --- | --- | --- | --- | --- | --- | --- | --- | --- |
|  | 1 | 2 | 3 | 1 | 2 | 3 | 1 | 2 | 3 |  |  |
| 0 | 24 | 22 | 24 | 126 | 125 | 127 | 111 | 110 | 111 | 126 | 1.00 |
| 2 | 26 | 23 | 26 | 131 | 130 | 132 | 105 | 105 | 106 | 131 | 1.00 |
| 4 | 26 | 28 | 27 | 137 | 138 | 138 | 107 | 106 | 107 | 137.67 | 0.58 |
| 6 | 26 | 20 | 23 | 145 | 141 | 142 | 105 | 102 | 101 | 142.67 | 2.08 |
| 8 | 27 | 23 | 26 | 146 | 145 | 146 | 99 | 99 | 110 | 145.67 | 0.58 |
| 10 | 24 | 25 | 27 | 149 | 150 | 148 | 96 | 97 | 96 | 149 | 1.00 |
| 12 | 32 | 30 | 32 | 154 | 154 | 155 | 93 | 94 | 95 | 154.33 | 0.58 |

**Table S7**

Comparison of methods for Cu^2+^ assays reported in literature

| **Sensing Material** | **Detection method** | **Detection limit** | **Real**  **samples** | **Cells imaging** | **Test**  **strips** | **Smartphone**  **detection** | **Refs.** |
| --- | --- | --- | --- | --- | --- | --- | --- |
| EDTA-Modified PANI/SWNTs Nanocomposite | Electrochemical | 1.4 µM | No | No | No | No | Sensors Actuators B Chem. 260 (2018) 331–338 |
| Indium tin oxide (ITO) | Electrochemical | 0.9988 mg/L | No | No | No | No | Sensors Actuators B Chem 248 (2017) 527–535 |
| Silver nanoparticles (AgNPs) | Electrochemical | 0.08 nM | Yes | No | No | No | Sensors Actuators B Chem 291 (2019) 164–169 |
| Ru-UiO-TPEA MOFs | Electrochemiluminescence  electrochemical | 0.26 nM  0.65 nM | Yes | No | No | No | Sensors Actuators B Chem 413 (2024) 135923 |
| Ni_5_P_4_/Ni_2_P/C/ITO | Electrochemical | 10 pM | Yes | No | No | No | Ceram. Int. 50 (2024) 18584–18593 |
| Silver nanoparticles (AgNPs) | Gas chromatography | 0.08 nM | Yes | No | No | No | J. Chromatog. A  1589 (2019) 116–121 |
| 2-hydroxyethyl methacrylate and bovine serum (IIP-HEMA-BSA) | Flame absorption atomic spectrometry | 1.1 μg L^−1^ | Yes | No | No | No | Talanta 202 (2019) 460–468 |
| Iron MNPs/stearic acid coated MNPs | Flame atomic absorption spectrometry | 0.15 mg/L | Yes | No | No | No | Measurement  186 (2021) 110108 |
| Coupling a peat mini-column to a flow system | Flame atomic absorption spectrometry | 3 μg L^−1^ | Yes | No | No | No | Anal. Chima. Acta  636 (2009)198–204 |
| [TBP] [PO_4_] IL-based dispersive | Liquid-liquid microextraction technique | 0.35 ppb | Yes | No | No | No | J. Food Compos. Anal.  89 (2020) 103457 |
| Free cyanide (HCN + CN^–^) | Liquid Chromatography-Tandem Mass Spectrometry | 0.07 μg/L | Yes | No | No | No | Anal. Chem. 87 (2015) 975–981 |
| 5-FAM-tripeptide | Fluorescence  Colorimetric | 49.7 nM | Yes | Yes | Yes | Yes | This work |

**Table S8**

Comparison of methods for glyphosate assays reported in literature

| **Sensing Material** | **Detection method** | **Detection limit** | **Real**  **samples** | **Cells imaging** | **Test**  **strips** | **Smartphone**  **detection** | **Refs.** |
| --- | --- | --- | --- | --- | --- | --- | --- |
| Polypyrrole (Ppy) | Electrochemical | 3.4 nM/L | No | No | No | No | Talanta 241 (2022) 123252 |
| Hollow fiber- pencil graphite | Electrochemical | 1.3 nM | Yes | No | No | No | Sensors Actuators B Chem. 272 (2018) 415–424 |
| Graphene oxide nanocomposite | Electrochemical | 11 µM | Yes | No | No | No | Int. J. Electrochem. Sci. 17 (2022) 221292 |
| Ti_3_C_2_Tx/Cu-BTC  nanocomposite | Electrochemical | 2.6 × 10^-14^ M | No | No | No | No | RSC Adv. 12 (2022) 5164–5172 |
| Polypyrrole nanotubes | Electrochemical | 1.94 ng/mL | No | No | No | No | Biosens. Bioelectron. 191 (2021) 113434 |
| Cu^0^-doped graphitic carbon quantum dots | Electrochemical | 2.02 ng/mL | Yes | No | No | No | Microchem. J. 200 (2024) 110294 |
| Trifluoroacetic anhydride and trifluoroethanol | Gas chromatography-mass spectrometry | 9 × 10^−12^ g | Yes | No | No | No | J. Anal.l Chem. 63 (2011) 371–375 |
| 9-fluorenylmethylchloroformate (FMOC-Cl) | High performance liquid chromatography | 0.01 mg/kg | Yes | No | No | No | J. Chromatogr. A 1502 (2017) 8–13 |
| 3,6-dimethoxy-9-phenyl-9H-carbazole-1-sulfonyl chloride (DPCS-Cl) | High performance liquid chromatography | 0.002 mg kg^−1^ | Yes | No | No | No | Anal. Methods 5 (2013) 6465–6472 |
| 9-fluorenylmethylchloroformate (FMOC-Cl) | High performance liquid chromatography | 5 ng/mL | Yes | No | No | No | J. Chromatogr. B 878 (2017) 3290-3296 |
| Gold coated magnetic nanoparticles (MNPs@Au) | Immunoassays | 20 ng•L^−1^ | No | No | No | No | Talanta 253 (2023) 123937 |
| Graphene quantum dots-silver nanoparticles | Luminescence | 9 ng mL^−1^ | Yes | No | No | No | Talanta 207 (2020) 120344 |
| Schiff base derivatives | Colorimetric | 1.78 µM  1.60 µM | Yes | No | No | No | J. Food Compos. Anal. 112 (2022) 104674 |
| 5-FAM-tripeptide | Fluorescence  Colorimetric | 66.3 nM | Yes | Yes | Yes | Yes | This work |
